# Chondrogenic Potential of Human Adipose-Derived Stem/Stromal Cells (hAD-MSCs) and Human Dental Pulp Stem/Stromal Cells (hDPSCs) Growing on a Poly L-Lactide-*Co*-Caprolactone Scaffold (PLCL)

**DOI:** 10.3390/cells15131168

**Published:** 2026-06-26

**Authors:** Julia K. Bar, Aleksandra Klimczak, Piotr G. Grelewski, Anna Lis-Nawara, Sandra Stamnitz, Tomasz Kowalczyk, Kinga Demska, Maria Paprocka, Hanna Gerber

**Affiliations:** 1 Department of Immunopathology and Molecular Biology, Wroclaw Medical University, Borowska 211, 50-556 Wroclaw, Poland; piotr.grelewski@umw.edu.pl (P.G.G.); anna.lis-nawara@umw.edu.pl (A.L.-N.); kinga.demska@umw.edu.pl (K.D.); 2 Laboratory of Biology of Stem and Neoplastic Cells, Hirszfeld Institute of Immunology and Experimental Therapy, Polish Academy of Sciences, R. Weigla 12, 53-114 Wroclaw, Poland; aleksandra.klimczak@hirszfeld.pl (A.K.); sandra.stamnitz@hirszfeld.pl (S.S.); maria.paprocka@hirszfeld.pl (M.P.); 3 Laboratory of Polymers and Biomaterials, Institute of Fundamental Technological Research (IPPT PAN), Polish Academy of Sciences, Adolfa Pawińskiego 5B St., 02-106 Warsaw, Poland; tkowalcz@ippt.pan.pl; 4 Department of Maxillofacial Surgery, Wroclaw Medical University, Borowska 213, 50-556 Wroclaw, Poland; hanna.gerber@umw.edu.pl

**Keywords:** stem/stromal cells, hDPSCs, hAD-MSCs, chondrocytes, chondrogenesis, poly(L-lactide-*co*-caprolactone) (PLCL), cartilage engineering

## Abstract

**Highlights:**

**What are the main findings?**

- Human dental pulp stem/stromal cells (hDPSCs) and adipose-derived stem/stromal cells (hAD-MSCs) grown on a poly(L-lactide-*co*-caprolactone) (PLCL) scaffold showed chondrogenic potential and an expression of chondrogenesis-related proteins similar to those of primary chondrocytes grown on PLCL.
- PLCL scaffold induces a microenvironment that enhances hDPSC, hAD-MSC, and chondrocyte adhesion, growth, and immunophenotypic stability.

**What are the implications of the main findings?**

- hDPSC/PLCL and hAD-MSC/PLCL showed good biocompatibility and might be considered as a bioimplant in cartilage engineering.
- Revealed chondrogenic potential of hDPSCs and hAD-MSCs, and their ability to synthesize a matrix, indicate that stem/stromal cells derived from different tissues are suitable for cartilage regeneration.

**Abstract:**

Cartilage engineering is a new therapeutic approach in regenerative medicine. This study explored the chondrogenic potential of human dental pulp stem/stromal cells (hDPSCs) and adipose-derived stem/stromal cells (hAD-MSCs) grown on a hydrolytically modified poly(L-lactide-*co*-caprolactone) (PLCL) electrospun scaffold in relation to the phenotype of primary chondrocytes on PLCL. The effects of PLCL scaffold on the biological features of hDPSC, hAD-MSC, and their chondrogenic differentiation and chondrocytes biology were evaluated via flow cytometry, immunochemistry, biochemistry, and RT–PCR. The results demonstrated that PLCL supported hDPSC, hAD-MSC, and chondrocyte viability and cellular attachment. The chondrogenic potential of hDPSCs and hAD-MSCs on PLCL scaffold was evidenced by the mRNA expression of the cartilage-specific genes. *Collagen type II (Col II)* and *aggrecan (Acan)* gene expression and their proteins significantly increased in chondrogenically differentiated hDPSCs and hAD-MSCs on PLCL compared with undifferentiated stem/stromal cells on PLCL. The phenotype of differentiated hDPSCs and hAD-MSCs was comparable to primary chondrocytes grown on PLCL. The results of this study showed that PLCL scaffold promoted chondrogenic differentiation of hAD-MSCs and hDPSCs toward chondrocytes with phenotypic similarities to native chondrocytes. The PLCL scaffold composition has a positive effect on hDPSC, hAD-MSC, and chondrocyte behavior, chondrogenic gene expression, and matrix protein synthesis.

## 1. Introduction

Injury of the articular cartilage can cause the degeneration of adjacent cartilage tissue, leading to osteoarthritis (OA), a degenerative disease characterized by bone and cartilage degradation [1]. During OA, chondrocytes release proteolytic enzymes that degrade collagen and proteoglycan, resulting in cartilage destruction [2]. Cartilage tissue repair is limited due to low chondrocyte proliferation and lack of blood vessels [3]. Many treatment approaches have been used for cartilage damage repair, such as microfractures, subchondral drilling, cartilage angioplasty, and nanofractures, which are mainly based on the stimulation of the bone marrow mesenchymal stem/stromal cells (BM-MSCs) [2,4]. Because surgical therapies are invasive and carry a risk of chronic infections and nerve damage, innovative therapies such as autologous chondrocyte implantation (ACI), matrix-assisted autologous chondrocyte implantation (MACI), or mesenchymal stem cells (MSCs) have been used for cartilage defect repair; however, clinical outcomes remain heterogeneous and unconvincing [3,4,5,6,7]. ACI and MACI have certain limitations associated with aging, dedifferentiation of chondrocytes into fibroblasts, and their low proliferative capacity, and these therapies can temporarily restore normal function in persons with cartilage degeneration, but joint replacement surgery may still be necessary [3,8]. Studies focusing on the use of MSCs in cartilage regeneration showed promising results [4,6,9]. MSCs have an unlimited therapeutic potential because they can differentiate into multiple cell lineages and have a greater proliferative capacity than chondrocytes and promote the formation of hyaline rather than fibrocartilage [10,11,12]. Adult MSCs can be isolated from almost every tissue of the body but have a different chondrogenic potential [4,6,13,14]. The most common source of MSCs used in cartilage repair is the bone marrow mesenchymal stromal cells (BM-MSC). Despite an established regenerative potential of BM-MSCs in animal models of cartilage defect similar to chondrocytes and also in several clinical trials, BM-MSCs have shown severe limitations, including an invasive harvesting procedure and a limited abundance [4,14,15,16]. Alternatively, human adipose-derived MSCs (hAD-MSCs) and human dental pulp stem cells (hDPSCs) showing similar properties to BM-MSCs, such as growth potential, biomarker expression profile, and differentiation capacity, are also used in cartilage repair [4,9,17,18]. There are studies that support chondrogenic potential of hDPSCs and suggest that dental pulp tissue may be a useful source of mesenchymal cells for use in articular cartilage repair [9,19]. The stromal vascular fraction (SVF) obtained through the enzymatic digestion of human adipose tissue or from mechanical micro-fragmented adipose tissue (MFAT) has been used for cartilage regeneration [20]. An in vitro study showed that hAD-MSCs and hDPSCs have a greater proliferation capacity than BM-MSCs and, after implantation into knee with OA, demonstrated regenerative potential [17,21,22,23,24,25,26]. However, only about 3% of MSCs suspension into synovial space remain in the knee joint in the short term, and few cells attach to the cartilage surface due to a lack of homing ability and poor engraftment and cellular retention at the injured sites, providing only transient pain reduction [27,28]. To overcome these limitations, different natural or synthetic biomaterials have been considered as platforms for cells in cartilage engineering [9,26,29,30]. Various scaffolds that promote chondrocyte growth and can support the adhesion, proliferation, and chondrogenic potential of MSCs have been investigated [9,12,14,24,31,32,33]. The mechanical and biological features of the scaffold should enable the creation of a microenvironment resembling native hyaline cartilage, rich in proteins such as *collagen type II (Col II)* and *aggrecan (Acan)*; free of *collagen type I (Col I)*, *collagen type X (Col X)*, and MMP-13 enzyme expression; and able to integrate with the native cartilage [9,32,33,34,35,36,37]. Hyaluronic acid, gelatin, chitosan, alginate, and collagen scaffolds are frequently used in cartilage repair due to their good biocompatibility, biodegradability, and biological properties supporting the adhesion, proliferation, and chondrogenic differentiation of stem cells [30,31,32,33,35,36]. Unfortunately, they have disadvantages related to rapid hydrolysis, which leads to a loss of properties suitable for the scaffold structure and low mechanical stability [29]. Polyglycolic acid (PGA), polylactic acid (PLLA), polystyrene, polycaprolactone (PCL), and poly-DL-lactic*-co-*glycolic acid (PLGA) scaffolds are more promising in cartilage regeneration because of their mechanical properties and a more suitable degradation rate in cartilage repair [38,39,40,41,42,43]. However, their bioactivity is not without limitations, as it presents the risk of rejection by the host organism and a lack of desirable biological properties [29,38]. Nevertheless, some of these materials have been approved by the Food and Drug Administration (FDA) for human clinical use [39,40,43]. Besides promising results of the use of bioscaffolds in cartilage engineering, it is not clear which properties of constructs determine successful cartilage regeneration, and their function in vitro and in vivo studies are poorly understood [29,32,33,37,38,44,45]. Recently, hybrid scaffolds for cartilage tissue engineering have been in development as 3D membranes (sponges), hydrogels, and nonwovens (nanofibers) [29,36,39,40,41,42,44,46,47,48]. Chondrotissue^®^ (Biotissue) is one of few hybrid scaffolds that have undergone clinical trials; five years of clinical trials have confirmed good outcomes, providing stable results with a future potential in hyaline cartilage regeneration [49]. There are data-recommended PLCL scaffolds combined with chondrocytes, BM-MSCs, or AD-MSCs, but not with hDPSCs for cartilage regeneration [50,51,52,53]. Our earlier results showed that hydrolytically modified electrospun PLCL scaffold characterized by high porosity and good mechanical properties has a better impact on hDPSCs’ osteogenic potential than a nonwoven hyaluronic acid (HYAFF-11™) scaffold [48]. An important advantage of co-polymer PLCL membrane is that they do not lose its strength in the aquatic environment, and its mechanical and biological durability is significantly better than that of the collagen substrate [52]. In this context, it would be interesting to investigate the effect of PLCL on hAD-MSCs and hDPSCs chondrogenesis. Growing evidence indicates that hybrid scaffolds provided the most promising results in articular cartilage regeneration; however, no scaffold has so far been developed that fully fulfills its proper roles in hyaline cartilage regeneration [29,40,44,50,53,54,55,56]. Despite the promising results showing that primary chondrocytes and chondrocytes derived from chondrogenic-differentiated BM-MSCs and hAD-MSCs on natural and synthetic scaffolds synthesize *Col II* in equal amounts up to now, there are not enough data to design a bioconstruct reflecting cartilage structure [57,58,59]. Given that the results regarding PLCL use for cartilage repair are limited, it seems important to analyze their biocompatibility with different cells in order to design a bioconstruct for cartilage regeneration that resembles native cartilage tissue. To our knowledge, there are no data on a comparison between chondrocytes derived from hAD-MSCs and hDPSCs upon chondrogenic differentiation on PLCL and primary chondrocytes cultured on a PLCL scaffold. Therefore, the aim of the study was to assess the biological behavior and chondrogenic potential of hDPSCs and hAD-MSCs growing on a PLCL scaffold using morphological, immunohistochemical, and molecular analyses in order to compare their chondrogenic potential with native chondrocytes growing on PLCL.

## 2. Materials and Methods

### 2.1. Biomaterial

#### Poly(L-lactide-*co*-caprolactone) (PLCL) Scaffold

In this study, an electrospun PLCL nanofiber scaffold (Purasorb 7015, Purac-Corbion, Amsterdam, The Netherlands), composed of 70% L-lactide and 30% caprolactone units, with an inherent viscosity of 1.5 dL/g was used as a platform for stem cells and chondrocytes. PLCL fabrication was performed according to the Good Manufacturing Practices certificate and used for the production of medical devices. Electrospinning was carried out in a Fluidnatek LE-50 chamber equipped with a humidity and temperature control module (Bioinicia, Valencia, Spain). The PLCL nanofiber scaffold was hydrolytically modified by immersing it in a 10% NaHCO_3_ aqueous solution for 4 days at 37 °C. The molecular weight and dispersion of the polymer were measured using gel permeation chromatography (GPC, Nexera LC-40, Shimadzu, Kyoto, Japan). The morphology of the PLCL nanofiber scaffold was assessed using scanning electron microscopy (SEM, JSM 6390 LV, Jeol, Tokyo, Japan) with 10 kV accelerating beam energy, as described in our earlier study. PLCL nanofiber scaffold has dispersity (Ð) 3.8, average fiber diameter between 3.1798 ± 0.7510 μm, and porosity achieve 73%. The average thickness of the PLCL nanofiber scaffold was close to 120 μm [60].

### 2.2. Methods of Stem/Stromal Cell and Chondrocyte Analysis

#### 2.2.1. Tissue Collection

Human dental pulp tissue was extracted from the third molars of three healthy volunteers (aged 14–28 years) undergoing routine tooth extraction. Adipose tissue was collected from the dermis of deceased organ donors (*n* = 3), aged 23–59 years, with the consent of the local Bioethics Committee (No. KB 792/2019). Cartilage fragments were removed from the apparently healthy regions of the internal femoral condyle from three patients by arthroscopic biopsy [61]. All study participants provided informed signed consent following a detailed explanation of the research procedure. All procedures regarding tissue collection and in vitro analysis of stem/stromal cells and chondrocytes were approved by the Bioethics Committee of the Medical University in Wroclaw, Poland (No. KB 513/2019 and No. KB 311/2024).

#### 2.2.2. Tissue Collection and hDPSC, hAD-MSC, and Chondrocyte Isolation and Culture

Human dental pulp tissue was used for stem/stromal cell isolation. hDPSCs were isolated and cultured according to the previously reported protocol [60]. The dental pulp tissue was separated from the teeth and digested for 1 h at 37 °C in a minimum essential medium (α-MEM; Gibco, Karlsruhe, Germany) containing 3 mg/mL of collagenase type I from *Clostridium histolyticum* (Sigma-Aldrich, St. Louis, MO, USA) and 4 mg/mL of dispase II (Gibco, Life Technologies, New York, NY, USA). Next, hDPSCs were cultured in flasks at 37 °C and 5% CO_2_ in α-MEM (Gibco) supplemented with 10% fetal bovine serum (FBS) (Gibco), 100 IU/mL penicillin, and 100 μg/mL streptomycin (Sigma-Aldrich). hAD-MSC isolation and culture were performed as described in detail [62]. hAD-MSCs were isolated from adipose tissue by digestion with 0.01% collagenase from *C. histolyticum* (Sigma-Aldrich) at 37 °C for 20 min of incubation and culture in α-MEM (IITE, Wroclaw, Poland), as previously described [62]. According to the recommendations of the International Society for Cellular Therapy (ISCT) [63] on the nomenclature and functional capacity of mesenchymal/stromal stem cells, hDPSCs and hAD-MSCs were investigated for the main features recommended by the ISCT to confirm stem cell origin, such as adherence to plastic, expression of specific cluster differentiation markers (CD), and tri-lineage differentiation potential for chondrogenesis, osteogenesis, and adipogenesis, as reported earlier [48,60,62]. Chondrocytes were isolated and cultured as previously described in detail [61,64]. The cartilage was digested in a growth medium (DMEM/F12) (Thermo Fisher Scientific, Rockford, IL, USA) containing 0.8 mg/mL collagenase II (Sigma-Aldrich) for 4 h at 37 °C, with 5% CO_2_ concentration. The isolated chondrocytes were cultured in a DMEM/F12 medium with 2 mM glutamax (Thermo Fisher Scientific), supplemented with 10% FBS, 100 IU/mL penicillin, and 100 μg/mL streptomycin (Sigma-Aldrich) [61]. All experiments were performed using hDPSCs, hAD-MSCs, and chondrocytes at the third passage (P3) in three replicates.

#### 2.2.3. hDPSC and hAD-MSC Phenotypic Characterization Using Flow Cytometry Analysis (FACS)

Phenotypes of hDPSCs and hAD-MSCs at passage 3 (P3) were characterized with flow cytometry using selected human-specific monoclonal antibodies detecting markers expressed by mesenchymal/stromal stem cells, as described in detail in our earlier studies [60,62]. Briefly, 2 × 10^5^ cells for each marker were resuspended in 50 µL of the PBS buffer and incubated with selected human-specific monoclonal antibodies, directly labeled with PE: CD73 (clone AD2), CD90 (clone 5E10), CD105 (clone 266), CD44 (clone 156-3C11), CD45 (clone HI30), CD31 (clone WM59), HLA ABC (clone G46-2.6), HLA DR (clone G46-6) (all from BD Pharmingen, San Jose, CA, USA), and Stro-1 (clone STRO-1) (Invitrogen, Thermo Fisher Scientific). Samples were analyzed using a flow cytometer (FACS Fortessa, Becton Dickinson, San Jose, CA, USA), and data were analysed using Flowing Software 2 (Flowing Software version 2.5.1., Turku, Finland).

#### 2.2.4. Multilineage Differentiation Potential of hDPSCs and hAD-MSCs

Tri-lineage differentiation of hDPSCs and hAD-MSCs into osteoblasts, chondroblasts, and adipocytes was performed according to the manufacturer’s protocol used in our previous experiments [60,62]. The differentiation of hDPSCs and hAD-MSCs into osteoblasts was confirmed with Alizarin Red S, chondrocytes with Alcian Blue, and adipocytes with Oil Red O staining [60,62].

#### 2.2.5. hDPSC, hAD-MSC, and Chondrocyte Seeding on a PLCL Nanofiber Scaffold

To assess the biological features of hDPSCs (*n* = 3), hAD-MSCs (*n* = 3), and chondrocytes (*n* = 3) growing on a PLCL scaffold, stem/stromal cells and chondrocytes were seeded at a density of 2 × 10^4^ cells/0.5 cm × 0.5 cm PLCL scaffold and cultured. hDPSCs and hAD-MSCs with the α-MEM medium for stem cells as described earlier [48,60] and chondrocytes with DMEM/F12 for 12 days. The stem/stromal cells and chondrocytes grown on the PLCL scaffold were observed using an Olympus IX73 inverted microscope (Olympus, Tokyo, Japan). After 12 days of hDPSC, hAD-MSC, and chondrocyte culturing on PLCL, the obtained bioconstructs were used for further analysis.

#### 2.2.6. Metabolic Activity of hDPSCs, hAD-MSCs, and Chondrocytes Grown on PLCL Assessed with the MTT Assay

The metabolic activity of hDPSCs, hAD-MSCs, and chondrocytes growing on the PLCL nanofibrous scaffold was analyzed using the MTT assay. The hDPSCs, hAD-MSCs, and chondrocytes were seeded at a density of 1 × 10^4^ on a 0.3 cm × 0.3 cm PLCL scaffold, cultured in 96-well plates, and incubated at 37 °C for 1, 3, and 7 days. Stem/stromal cells and chondrocytes cultured without the scaffold served as controls. Next, the scaffold was removed, and 200 µL of 3-(4,5-dimethylthiazol-2-yl)-2,5-diphenyltetrazolium bromide (Sigma-Aldrich) was added to the plates with the hDPSCs, hAD-MSCs, and chondrocytes before incubation at 37 °C for 4 h. After 4 h of incubation, the formazan crystals were dissolved by adding 100 µL of acidic isopropanol (38% HCl in 99.7% isopropanol) [60]. Absorbance was measured at 570 nm using a GloMax^®^ Discover multimode microplate reader (Promega, Madison, WI, USA). The experiments were performed in three replicates for each group of cells, and data were calculated as means ± *SD*.

#### 2.2.7. Morphological Features of hDPSCs, hAD-MSCs, and Chondrocytes Grown on a PLCL Scaffold

The morphological features of chondrocytes on PLCL and hDPSC, hAD-MSC on a PLCL scaffold before and after chondrogenic differentiation was examined. For the analysis of the morphological features of the hDPSCs, hAD-MSCs, and chondrocytes, PLCL/bioconstructs were fixed in 10% formalin solution (Merck, Saint Louis, MO, USA) for 20 min and stained with hematoxylin and eosin (H&E) according to the manufacturer’s protocol. Afterwards, the morphological features and location of cells on the bioconstructs were examined using a BX51 microscope (Olympus) [48,60].

#### 2.2.8. Cytoskeleton Analysis in hDPSCs, hAD-MSCs, and Chondrocytes Grown on a PLCL Scaffold

The cytoskeleton of hDPSCs, hAD-MSCs, and chondrocytes grown on a PLCL scaffold was determined with actin visualization using phalloidin staining. F-actin expression in hDPSCs and hAD-MSCs grown on PLCL before and after chondrogenic differentiation was evaluated and compared. Stem/stromal cells and chondrocytes on the PLCL scaffold were fixed for 15 min at room temperature (RT) in 4% paraformaldehyde. Next, the cells/scaffolds were treated with 0.1% Triton™ X-100 for 15 min and washed three times with PBS. hDPSCs/PLCL, hAD-MSCs/PLCL, and chondrocytes/PLCL constructs were incubated with Alexa Fluor 488-Phalloidin (Thermo Fisher Scientific) (dilution of 5 µL of methanol stock solution in 200 µL of PBS) for 40 min. In the next step, the cells/PLCL constructs were stained with 4′,6-diamidino-2-phenylindole (DAPI) for nuclei visualization. F-actin expression on cells/PLCL and cell distribution on the scaffold were estimated using a BX61 fluorescence microscope (Olympus), as described earlier in our studies [48,60].

#### 2.2.9. Chondrogenic Differentiation of hDPSCs and hAD-MSCs on a PLCL Scaffold

hDPSCs (*n* = 3) and hAD-MSCs (*n* = 3) were seeded at a density of 4 × 10^3^ cells per cm^2^ on PLCL and cultured in complete α-MEM medium until 90% confluence was reached. Next, the culture medium was removed, and the hDPSCs/PLCL and hAD-MSCs/PLCL constructs were maintained in an hMSC chondrogenic induction medium, serum-free kit (Provitro, Berlin, Germany), and cultured for 28 days according to our established protocol [60]. Undifferentiated hDPSCs and hAD-MSCs grown on PLCL served as controls. The induction medium was changed three times a week.

### 2.3. Evaluation of the Chondrogenic Potential of hDPSCs and hAD-MSCs on a PLCL Scaffold

#### 2.3.1. Alcian Blue Staining

The functional status of hDPSCs (*n* = 3) and hAD-MSCs (*n* = 3) on PLCL differentiated into chondrocytes was confirmed with Alcian Blue (Abcam Inc., Cambridge, UK) staining to show the rate of cellular matrix deposition. ECM was also analyzed in chondrocytes grown on PLCL. Differentiated stem/stromal cells on the PLCL scaffolds and chondrocytes on PLCL were washed with PBS, fixed in a 10% formalin solution (Merck), washed again with PBS, and incubated with Alcian Blue for 30 min in a dark chamber at RT [60]. Finally, the bioconstructs with differentiated and undifferentiated stem cells and with chondrocytes were washed once in running tap water and twice in distilled water and analyzed using an Olympus IX73 microscope (Olympus). Undifferentiated hDPSCs and hAD-MSCs grown on PLCL served as negative controls [60].

#### 2.3.2. Antibodies

For immunohistochemical staining, the following antibodies were used: anti-collagen I (*Col I*) (mouse monoclonal, clone (*Col I*), dilution 1:2000 (Thermo Fisher Scientific); anti-collagen II (*Col II*) (mouse monoclonal, clone M2139), dilution 1:100 (Thermo Fisher Scientific); anti-Sox9 (mouse monoclonal, clone 1B11), 1:200 dilution (Thermo Fisher Scientific); anti-collagen X (mouse monoclonal *Col X*), dilution 1:1000 (Sigma-Aldrich); and anti-aggrecan (mouse monoclonal, clone 969D4D11), dilution 1:1000 (Invitrogen, Thermo Fisher Scientific).

#### 2.3.3. Immunohistochemical Staining (IHC)

Immunohistochemical staining of chondrogenic proteins was performed on specimens before and after hDPSC and hAD-MSC chondrogenic differentiation on PLCL and chondrocyte growth on PLCL using the Universal Dako REAL EnVision Detection System, Peroxidase/DAB+, Rabbit/Mouse (Dako, Copenhagen, Denmark), and the following primary antibodies detected *Col I*, *Col II*, *Col X*, *Acan*, and *Sox9*. hDPSCs/PLCL, hAD-MSCs/PLCL, and chondrocytes/PLCL specimens were fixed in a 4% formalin solution (Merck) for 15 min. Next, endogenous peroxidase reactivity was blocked with the Dako REAL Peroxidase Blocking Solution (Dako), after which the bioconstruct specimens were incubated with primary antibodies overnight at 4 °C. After washing with a 0.1 M Tris buffer, pH = 7.4 (TBS), the scaffold specimens were incubated with Dako REAL EnVision/HRP, Rabbit/Mouse (Dako) for 30 min at RT. The antigen-antibody reaction was visualized using DAB (3,3 diaminobenzidine) (Dako) as a chromogen for 4 min at RT. The sections were counterstained with hematoxylin. An incubation buffer (TBS) without primary antibodies was used as a negative control [60].

#### 2.3.4. Assessment and Interpretation of Immunohistochemical Staining

Expression of the analyzed proteins in undifferentiated and chondrogenic differentiated hDPSCs and hAD-MSCs located on PLCL and in chondrocytes on PLCL scaffold was assessed semi-quantitatively, taking into account staining intensity and the number of cells showing immunoreactivity for the analyzed proteins, such as *Col I*, *Col II*, *Col X*, *Acan*, and *Sox9*. The percentage of immunopositive cells for the analyzed proteins was determined by counting 1000 cells in a randomly selected field of the hDPSCs/PLCL, hAD-MSCs/PLCL, and chondrocytes/PLCL constructs using an Olympus BX51 microscope (Olympus). Specimens with over 5% of immunopositive cells were considered as positive. The intensity score was based on the color of the reaction, where no color = no immunostaining, light yellow color = weak (+), medium brown color = moderate (++), and brown color = strong (+++) immunostaining [48,60].

#### 2.3.5. Quantitative Polymerase Chain Reaction (qPCR) for Chondrogenic Gene Expression

To evaluate the expression of the chondrogenic marker genes—*Sox9*, *collagen type I (Col I)*, *Col II*, *collagen type X (Col X)*, and *Acan*—total RNA was extracted from hAD-MSCs-PLCL, hDPSCs-PLCL, and chondrocytes-PLCL under both control conditions and after chondrogenic induction. Cells derived from three independent donors were cultured on a PLCL scaffold for each cell type, and the data were presented as the mean value. RNA was isolated using the RNeasy Plus Mini Kit (Qiagen, Hilden, Germany), following the manufacturer’s protocol. RNA concentration and purity were assessed spectrophotometrically. For each sample, 1 μg of total RNA was reverse-transcribed into cDNA using the RevertAid First Strand cDNA Synthesis Kit (Thermo Fisher Scientific). Quantitative PCR was performed using the ViiA 7 Real-Time PCR System (Applied Biosystems, Foster City, CA, USA) and Power SYBR Green PCR Master Mix (Life Technologies, Warrington, UK). Gene-specific primers targeting *Sox9*, *Col II*, *Acan*, *Col I*, and *Col X* were used to evaluate chondrogenic differentiation. Reactions were carried out in triplicate using the following thermal profile: initial denaturation at 95 °C for 10 min, followed by 40 cycles of 95 °C for 15 s, annealing at 60 °C for 1 min, and extension at 72 °C for 40 s. Gene expression levels were normalized to the housekeeping gene *β-2 microglobulin (B2M)*, and relative expression was calculated using the 2^−ΔΔCt^ method. The results are presented as fold change in expression. Primer sequences are listed in Table 1.

#### 2.3.6. Statistical Analysis

The data are presented as a mean ± 95% CI. The confidence intervals were calculated using Wald’s method. The statistical differences between experimental groups in viability and chondrogenic-related proteins were determined using a Student’s *t*-test. Statistical analysis was performed using Statistica version 13 (TIBCO Software Inc., Palo Alto, CA, USA). For the comparison of chondrogenesis-related gene expression, a one-way analysis of variance (one-way ANOVA) with Dunnett’s test for multiple comparison procedures was used. Statistical analysis was performed using GraphPad Prism version 7 (GraphPad Software, Boston, MA, USA). Statistical significance was considered for *p*-values < 0.05.

## 3. Results

### 3.1. Immunophenotypic Identification of Human MSCs

The antigen profile of hDPSC and hAD-MSC cells was analyzed using flow cytometry. The results demonstrated that MSCs derived from two different tissue sources showed a similar morphology in in vitro culture (Figure S1A,B) and were positive for surface markers such as CD73, CD105, CD90, CD44, as well as HLA ABC and were negative for surface markers such as CD45, CD31, CD34, and HLA DR. hDPSCs and hAD-MSCs expressed the STRO-1 marker in a similar percentage of cells (Figure S1C,D). The antigen profile of hDPSCs and hAD-MSCs was similar, as shown in Figure S1C,D.

### 3.2. Differentiation Capacity of MSCs Derived from Different Tissues

Figure S2 presents hDPSC and hAD-MSC tri-lineage differentiation potential. hDPSCs and hAD-MSCs differentiated into osteoblasts revealed strong Alizarin S staining (Figure S2A,B), and both types of cells differentiated into chondrocytes revealed a similar pattern of Alcian Blue staining, as shown in Figure S2C,D. The adipogenic differentiation capacity of hDPSCs and hAD-MSCs was confirmed with Oil Red O staining, and MSCs derived from both tissues had comparable adipogenic capacity (Figure S2E,F).

### 3.3. Metabolic Activity of Cells Grown on PLCL Membrane

**Figure 1:**
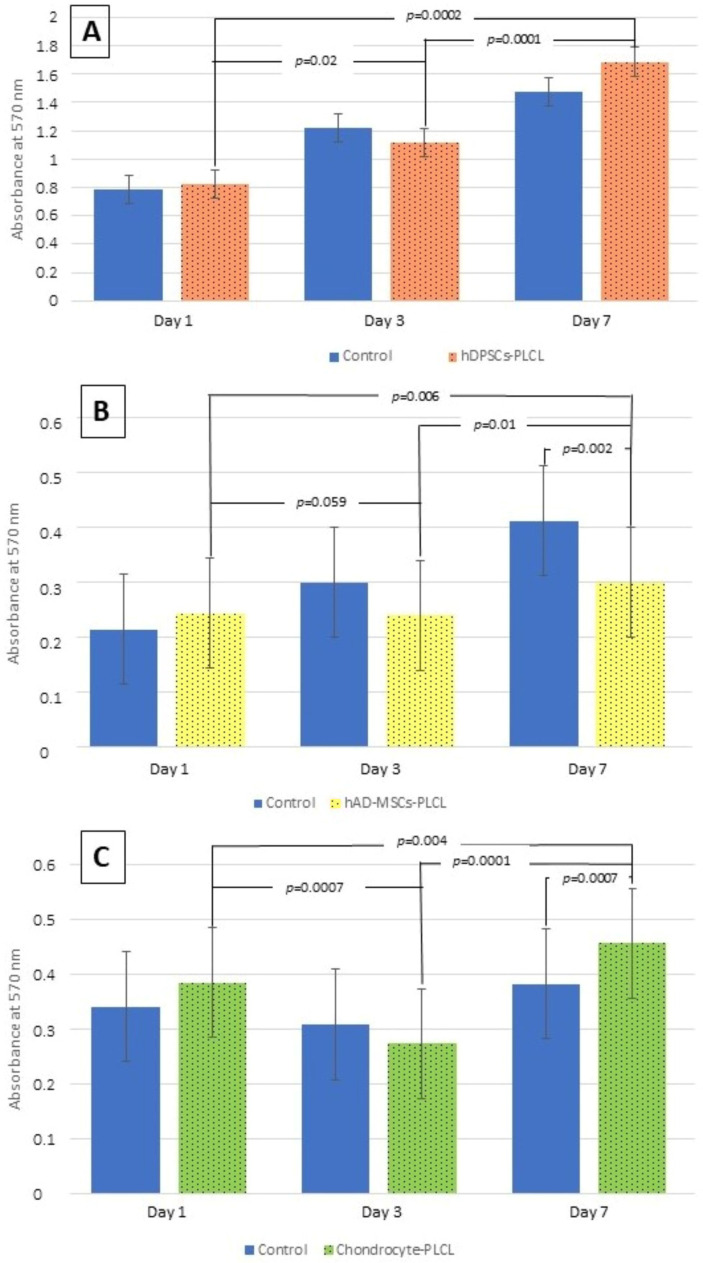
Metabolic activity diagram of hDPSCs, hAD-MSCs, and chondrocytes grown on PLCL, analyzed by MTT assay. (**A**) MTT assay showed significantly higher metabolic activity of hDPSCs on PLCL on days 3 and 7 than on day 1 (*p* = 0.02, *p* = 0.0002, respectively) and on day 7 than on day 3 (*p* = 0.0001). (**B**) Significantly lower metabolic activity of hAD-MSCs was found between control group and hAD-MSCs on PLCL on day 7 (*p* = 0.002) and higher in hAD-MSCs grown on PLCL on day 7 than on days 1 and 3 (*p* = 0.006, *p* = 0.02, respectively). (**C**) Metabolic activity of chondrocytes was significantly higher in control group compared to chondrocytes on PLCL on day 7 (*p* = 0.0007) and on day 7 than on days 3 and 1 (*p* = 0.0001, *p* = 0.004) and on day 1 than on day 3 (*p* = 0.0007). Error bars indicate “mean ± *SD*”. Statistically significant differences: *p* < 0.05.

In order to test the cytotoxicity of the PLCL membrane on hDPSCs, hAD-MSCs, and chondrocytes, an MTT analysis was performed for 1, 3, and 7 days of culture. The metabolic activity of hDPSCs, hAD-MSCs, and chondrocytes grown on PLCL membrane, as assessed with the MTT assay, showed good biocompatibility of PLCL. The MTT analysis after 1, 3, and 7 days demonstrated the nontoxic influence of PLCL membrane on cell growth. As presented in Figure 1A, no statistical differences in hDPSC metabolic activity were observed between the control group and hDPSCs on PLCL at days 1, 3, and 7 of culture. The metabolic activity of hDPSCs grown on PLCL showed differences between days 1 and 3 and 7 (*p* = 0.02, *p* = 0.0002) and between days 3 and 7 (*p* = 0.001) (Figure 1A). hAD-MSCs grown on PLCL showed significantly lower metabolic activity than in the control group on day 7 (*p* = 0.002) (Figure 1B). The metabolic activity of hAD-MSCs on PLCL on day 1 was comparable with day 3 (*p* = 0.059) and was significantly higher at day 7 than at day 1 (*p* = 0.006) and day 3 (*p* = 0.02) (Figure 1B). The metabolic activity of chondrocytes on PLCL on days 1 and 3 was comparable to that of the control group, but was significantly higher than in the control group on day 7 (*p* = 0.0007) (Figure 1C). The metabolic activity of chondrocytes on PLCL showed significant differences between days 1 and 3 (*p* = 0.0007) and day 7 (*p* = 0.004) and between days 3 and 7 (*p* = 0.0001) (Figure 1C).

### 3.4. Distribution and Morphological Features of Cells Grown on an Electrospun Nanofibrous PLCL Scaffold

**Figure 2:**
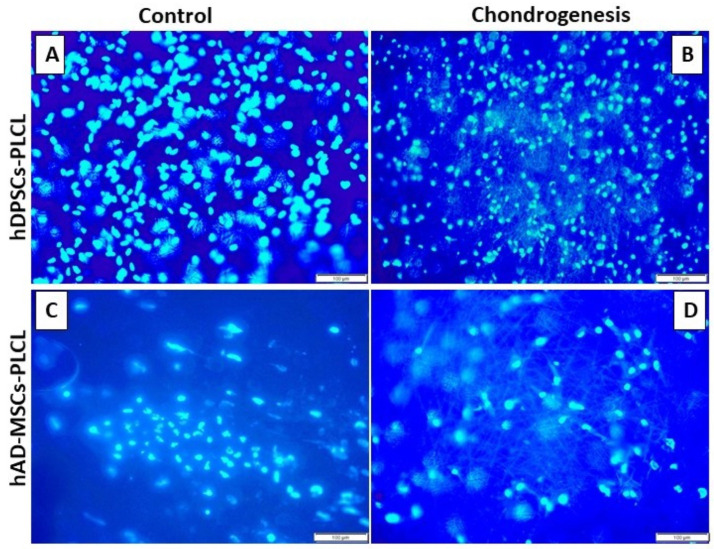
The distribution of hDPSCs and hAD-MSCs on PLCL scaffold. (**A**) High density of undifferentiated hDPSCs and (**B**) differentiated hDPSCs. The distribution of hAD-MSCs on PLCL (**C**) before and (**D**) after chondrogenesis showed a high cell density, visible by cell nuclei counterstained with DAPI. Nuclei counterstained with DAPI are shown in blue. Scale bars: 100 µm.

**Figure 3:**
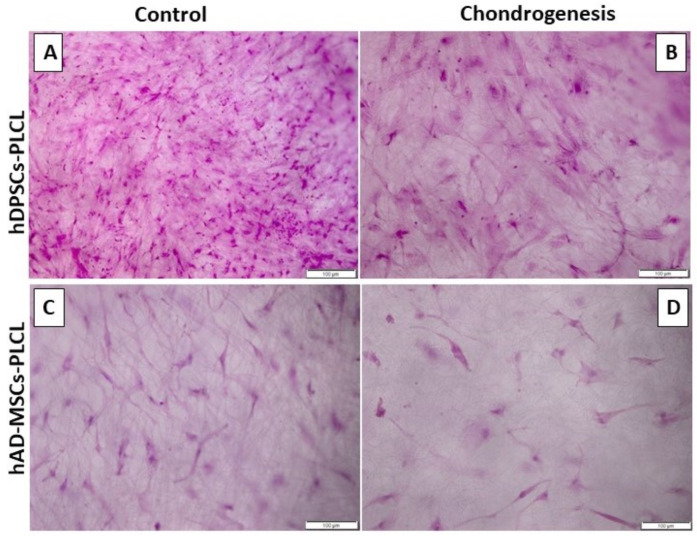
The morphology of hDPSCs and hAD-MSCs grown on PLCL scaffold before and after chondrogenic differentiation. (**A**) Undifferentiated hDPSCs showed a fibroblast-like shape, and (**B**) after chondrogenesis, the differentiated cells revealed changes in morphological features toward chondrocytes. (**C**) Undifferentiated hAD-MSCs showed a fibroblast-like shape, and (**D**) after chondrogenesis, the differentiated cells revealed changes in morphological features toward chondrocytes (H&E). Scale bars: 100 µm.

**Figure 4:**
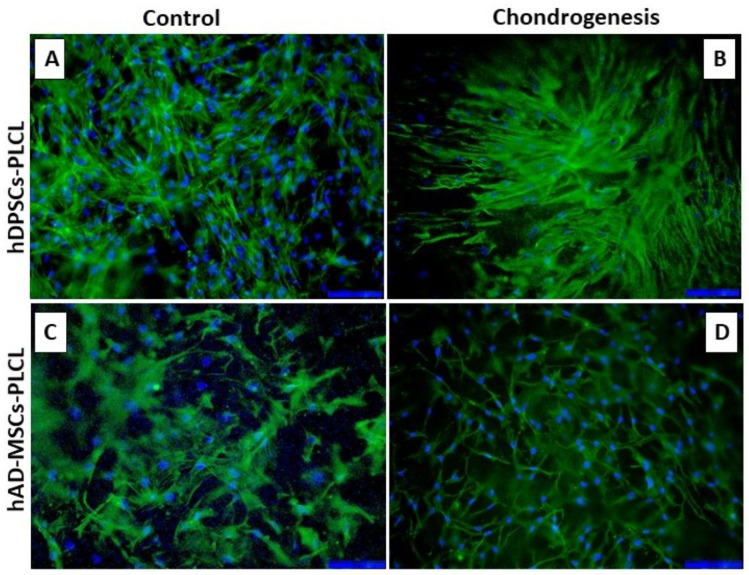
F-actin expression in hDPSCs and hAD-MSCs grown on PLCL scaffold. Immunofluorescence staining of F-actain expression in hDPSCs grown on PLCL (**A**) before and (**B**) after chondrogenesis showed a similar pattern of F-actin expression in undifferentiated and chondrogenically differentiated hDPSCs. Detection of F-actin expression in hAD-MSCs grown on PLCL (**C**) before and (**D**) after chondrogenesis using Alexa Fluor 488-conjugated phalloidin. Nuclei counterstained with DAPI are shown in blue. Scale bars: 100 µm.

**Figure 5:**
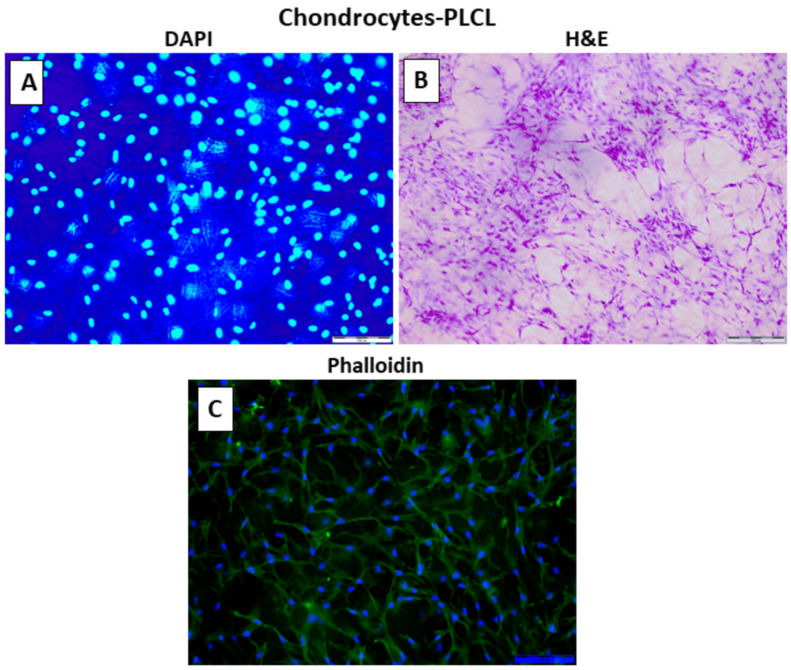
Representative image of chondrocytes grown on PLCL displaying (**A**) high density of chondrocytes on PLCL. Nuclei counterstained with DAPI are shown in blue. (**B**) Morphological features of chondrocytes on PLCL. (**C**) Chondrocytes showed F-actin expression detected by phalloidin staining. Scale bars: (**A**,**C**) 100 µm and (**B**) 200 µm.

Distribution, morphology, and F-actin expression of hDPSCs and hAD-MSCs before and after chondrogenesis on PLCL are shown in Figure 2A–D, Figure 3A–D and Figure 4A–D. Chondrocyte features on electrospun PLCL scaffolds are presented in Figure 5A–C. The image revealed that undifferentiated hDPSCs and hAD-MSCs (Figure 2A,C), differentiated hDPSCs and hAD-MSCs grown on the PLCL scaffold (Figure 2B,D), and chondrocytes on PLCL (Figure 5A) demonstrated good adhesion to the fibrous structure of the scaffolds. Cells were uniformly distributed along the scaffold, but the majority of stem/stromal cells before (Figure 2A–C) and after chondrogenic differentiation (Figure 2B–D) and chondrocytes (Figure 5A) grew in the central part of the scaffold, rather than at its edges. The morphological features of undifferentiated and differentiated hDPSCs, hAD-MSCs on PLCL are presented in Figure 3A–D, and chondrocytes on PLCL scaffolds are shown in Figure 5B. The morphological characteristics of undifferentiated hDPSCs and hAD-MSCs exhibit fibroblast-like morphology (Figure 3A,C). Chondrocytes derived from differentiated hDPSCs and hAD-MSCs (Figure 3B,D) showed many common morphological features with native chondrocytes (Figure 5B). The cells showed rounded morphology, which is an essential chondrocyte characteristic, and a tendency to aggregate. A majority of the differentiated cells presented an oval shape, large nuclei with dense chromatin, and stable cytoskeleton. Phalloidin staining of undifferentiated and chondrogenic differentiated hDPSCs and hAD-MSCs on PLCL showed comparable levels of F-actin expression before (Figure 4A,C) and after cell differentiation (Figure 4B,D). However, as presented in Figure 5C, F-actin fibers staining predominated in primary chondrocytes on PLCL compared to differentiated hDPSCs and hAD-MSCs (Figure 4B,D).

### 3.5. In Vitro Pro-Chondrogenic Potential of hDPSCs and hAD-MSCs Grown on a PLCL Scaffold

#### 3.5.1. Proteoglycan Matrix Detection by Alcian Blue Staining

**Figure 6:**
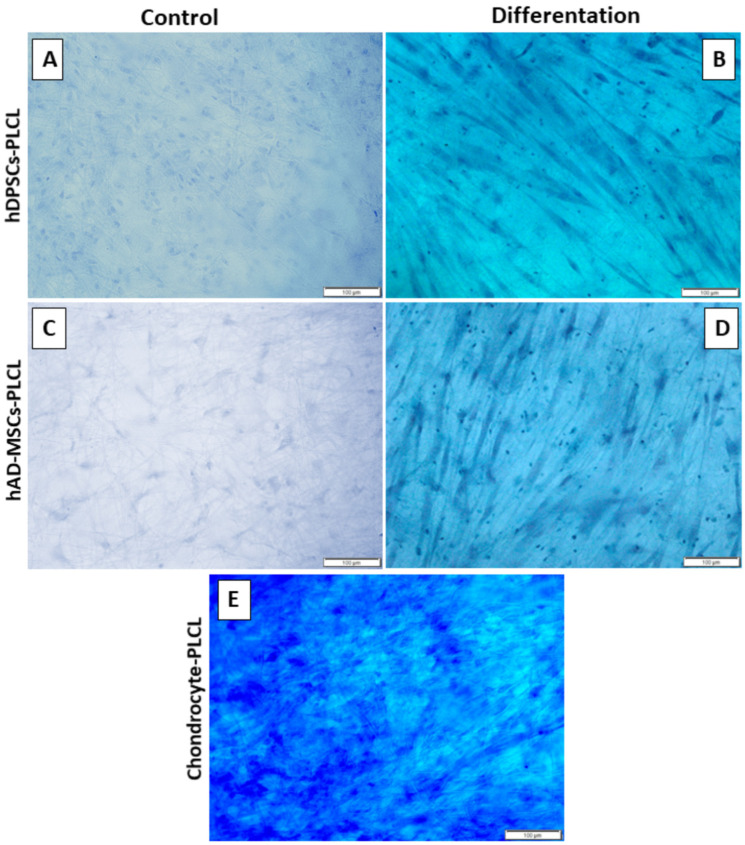
Representative images displaying Alcian Blue staining of chondrogenically differentiated hDPSCs and hAD-MSCs on PLCL and chondrocytes grown on PLCL. (**A**) Negative Alcian Blue staining in undifferentiated hDPSCs. (**B**) Higher accumulation of GAG found in hDPSC differentiation. (**C**) No GAG accumulation in undifferentiated hAD-MSCs. (**D**) Differentiated hAD-MSCs showed strong positive Alcian Blue staining. (**E**) Primary chondrocytes showed very high matrix deposition. Scale bars: 100 µm.

Histological examination of undifferentiated hDPSCs-PLCL and hAD-MSCs-PLCL and differentiated into chondroblasts showed the differences in GAG production (Figure 6A–D). The proteoglycan matrix synthesized by differentiated hDPSCs and hAD-MSCs was positively stained with Alcian Blue (Figure 6B,D). Chondrogenically differentiated hDPSCs and hAD-MSCs (Figure 6B,D) had a higher GAG content than undifferentiated stem cells (Figure 6A,C), but lower than native chondrocytes grown on PLCL (Figure 6E). No remarkable differences in the cartilage matrix formation were observed between hDPSCs and hAD-MSCs on PLCL after chondrogenesis.

#### 3.5.2. Chondrogenesis-Related Protein Expression

**Figure 7:**
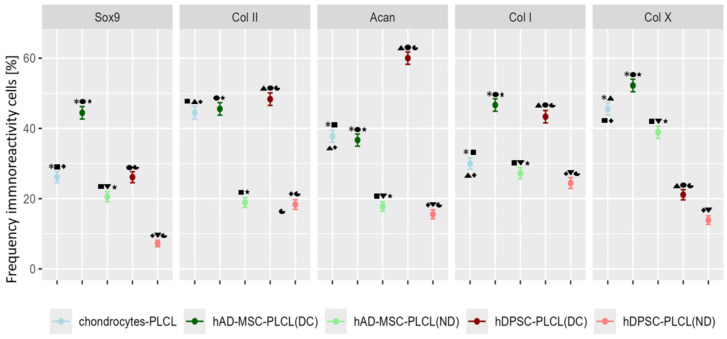
Comparison of chondrogenesis-related proteins expression in hDPSCs, hAD-MSCs, and chondrocytes grown on PLCL scaffold. Significantly higher expression of *Sox9*, *Col I*, *Col II*, *Col X*, and *Acan* was found in ★ hAD-MSCs-PLCL (DC) than hAD-MSCs-PLCL (ND), in 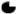 hDPSCs-PLCL (DC) than hDPSC-PLCL (ND), in ⯀ chondrocytes-PLCL than hAD-MSCs-PLCL(NC), in ⯁ chondrocytes-PLCL than hDPSCs-PLCL (ND), in ⬤ hAD-MSCs-PLCL (DC) than hDPSCs-PLCL(CD) (*p* < 0.05). ⯆ hAD-MSCs-PLCL(ND) revealed higher expression of *Sox9*, *Col I*, *Col X*, and *Acan* than hDPSCs-PLCL(ND) (*p* < 0.05). ✱ Chondrocytes-PLCL exhibited higher *Sox9*, *Col I*, and *Col X* expression than hAD-MSCs-PLCL(DC) (*p* < 0.05). ⯅ hDPSCs-PLCL(DC) showed higher *Col I*, *Col II*, *Col X*, and *Acan* expression compared with chondrocytes-PLCL (*p* < 0.05). ND-undifferentiated stem/stromal cells, DC-chondrogenic differentiated. Data are presented as means ± *SD*. Statistical significance: *p* < 0.05.

The expression of *Col I*, *Col II*, *Col X*, *Acan*, and *Sox9* in hDPSCs and hAD-MSCs grown on PLCL was analyzed before and after chondrogenic differentiation and in native chondrocytes on PLCL. As presented in Figure 7, a comparative analysis showed significant differences in *Col I*, *Col II*, *Col X*, *Acan*, and *Sox9* protein expression between undifferentiated and differentiated stem/stromal cells and between the analyzed groups of cells. All analyzed proteins revealed significantly higher expression in chondrogenically differentiated hAD-MSCs and hDPSCs than in undifferentiated hAD-MSCs and hDPSCs (*p* < 0.05) (Figure 7). A comparison of chondrogenic proteins between undifferentiated hAD-MSCs/PLCL and hDPSCs/PLCL showed higher expression of *Col I*, *Col X*, *Sox9*, and *Acan* in hAD-MSCs than in hDPSCs (*p* = 0.05) (Figure 7). No differences were observed in *Col II* expression between hAD-MSCs and hDPSCs (*p* > 0.05). A comparison between undifferentiated hAD-MSCs/PLCL, hDPSCs/PLCL, and chondrocytes/PLCL constructs revealed that the expression of *Col I*, *Col II*, *Col X*, *Acan*, and *Sox9* was significantly higher (*p* < 0.05) in chondrocytes/PLCL than in undifferentiated hAD-MSCs and hDPSCs on the PLCL membrane, as shown in Figure 7. A comparative analysis of chondrogenic proteins between differentiated hAD-MSCs and hDPSCs on PLCL and between chondrocytes grown on PLCL showed significant differences. The expression of *Col I*, *Col X*, and *Sox9* was significantly higher in chondrogenically differentiated hAD-MSCs on PLCL than in hDPSCs-PLCL (*p* < 0.05), whereas the expression of *Col II* and *Acan* was significantly higher in differentiated hDPSCs/PLCL than in hAD-MSCs (*p* < 0.05) (Figure 7). *Col I*, *Col X*, and *Sox9* expression was significantly higher in chondrocytes derived from chondrogenically differentiated hAD-MSCs/PLCL than in native chondrocytes grown on PLCL (*p* < 0.05) (Figure 7), but the expression of *Col II* and *Acan* showed no differences between differentiated hAD-MSCs and native chondrocytes grown on PLCL (*p* > 0.05). The expression of chondrogenic proteins, such as *Col I*, *Col II*, and *Acan*, was higher in differentiated hDPSCs on PLCL than in native chondrocytes on PLCL (*p* < 0.05), but *Col X* expression was higher in chondrocytes/PLCL than in hDPSCs/PLCL (*p* < 0.05) (Figure 7). No differences in *Sox9* expression were observed between hDPSCs/PLCL and native chondrocytes/PLCL (*p* > 0.05) (Figure 7).

**Figure 8:**
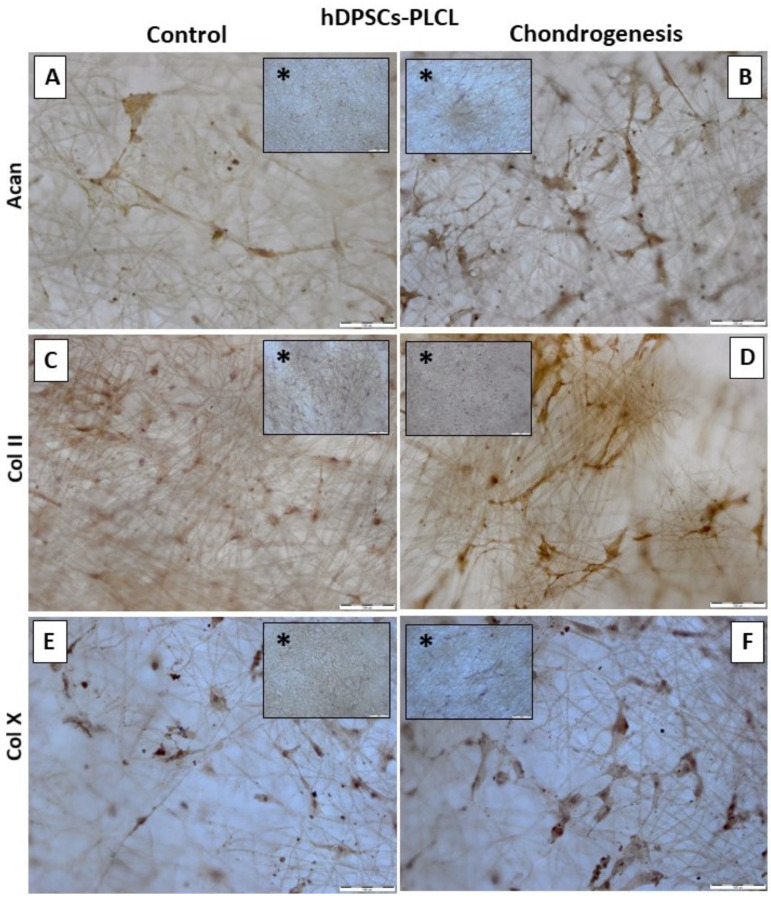

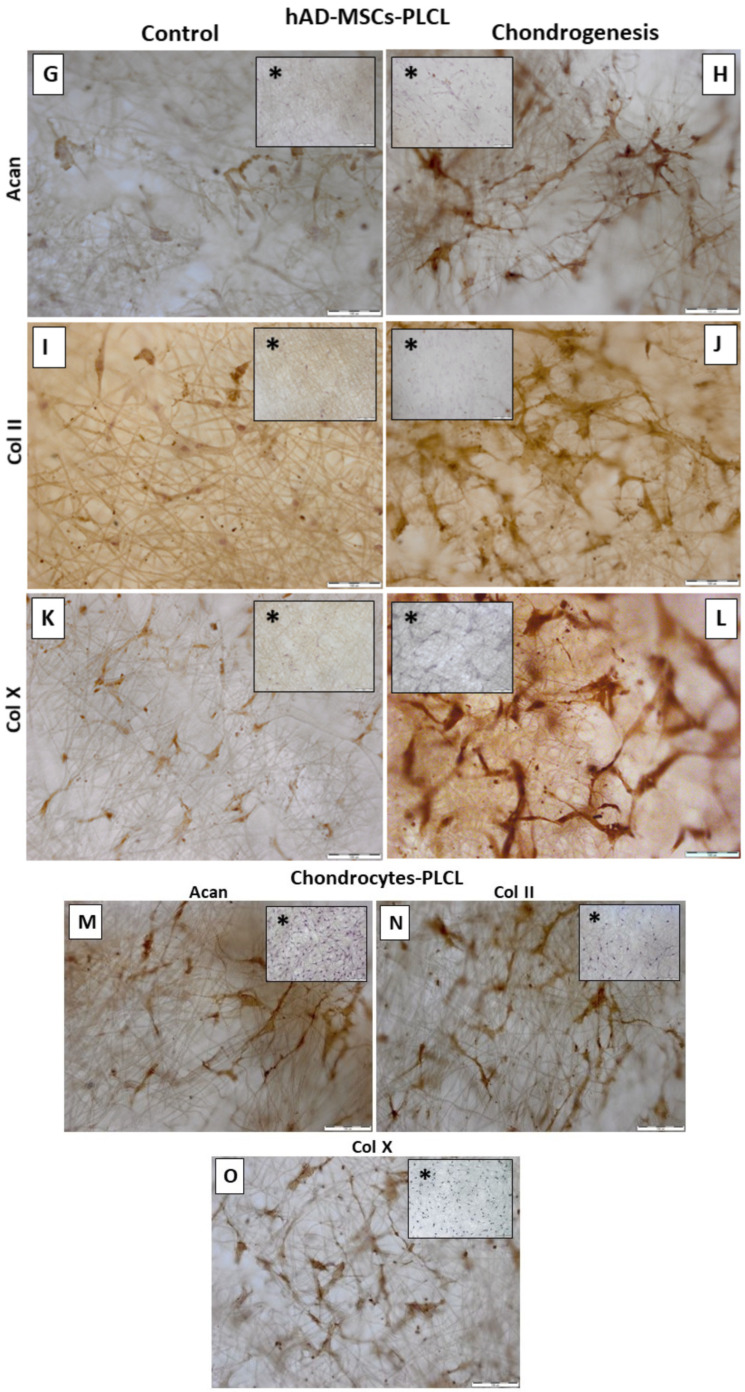
Comparison of chondrogenesis-related proteins expressed by hDPSCs and hAD-MSCs grown on PLCL nonfibrous scaffold before and after chondrogenic differentiation and by chondrocytes grown on PLCL (**A**–**O**). Undifferentiated hDPSCs-PLCL with (**A**) low *Acan*, (**C**) *Col II*, and (**E**) pattern of *Col X* expression. Chondrogenic differentiated hDPSCs-PLCL (**B**) with high *Acan* immunopositivity, (**D**) strong *Col II* diffuse immunoreactivity, and (**F**) strong immunopositivity for *Col X* found in a high number of differentiated hDPSCs on PLCL. Undifferentiated hAD-MSCs-PLCL (**G**) with low *Acan* and (**I**) *Col II* and (**K**) *Col X* expression limited to several immunopositive hAD-MSCs. The majority of chondrogenically differentiated hAD-MSCs-PLCL showed moderate immunopositivity for (**H**) *Acan* and (**J**) *Col II* staining and (**L**) strong immunostaining for *Col X*. (**M**) *Acan* expression was found in many chondrocytes on PLCL. (**N**) *Col II* immunopositivity was observed in the majority of chondrocytes on PLCL. (**O**) Chondrocytes on PLCL showed moderate expression of *Col X* (EnVision technique). * indicates a control group. Scale bars: 100 μm.

The analyzed cartilage-related proteins, after chondrogenic differentiation, hDPSCs and hAD-MSCs located on PLCL constructs showed different ranges of immunostaining and immunoenzymatic reaction intensities (Figure 8A–O). As seen in a set of selected microscopic images showing cartilage-related proteins expressed by chondrocytes derived from hDPSCs and hAD-MSCs, this displayed variable expression of chondroblast/chondrocyte biomarkers such as *Sox9*, *Col I*, *Col II*, *Col X* and *Acan*. The expression of *Acan*, *Col II*, and *Col X* in differentiated hDPSCs/PLCL (Figure 8B,D,F) and hAD-MSCs/PLCL (Figure 8H,J,L) was higher than in undifferentiated hDPSCs/PLCL (Figure 8A,C,E) and hAD-MSC/PLCL (Figure 8G,I,K), but comparable with the expression of *Acan*, *Col II*, and *Col X* in native chondrocytes grown on PLCL (Figure 8M–O).

#### 3.5.3. Chondrogenesis-Related Gene Expression

**Figure 9:**
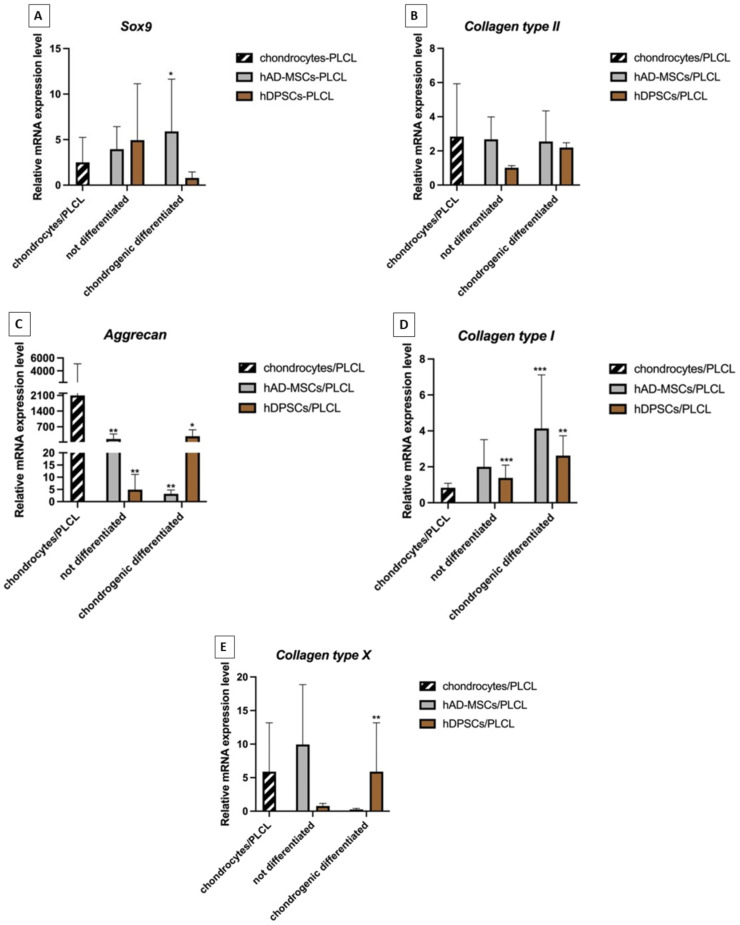
Relative mRNA expression of chondrogenic marker genes in hAD-MSCs, hDPSCs, and chondrocytes under control conditions and following chondrogenic induction. Expression levels of (**A**) *Sox9*, (**B**) *Coll II*, (**C**) *Acan*, (**D**) *Coll I*, and (**E**) *Coll X* were assessed using quantitative real-time PCR in three different cell types cultured on PLCL: human adipose tissue-derived mesenchymal stem cells (hAD-MSCs), human dental pulp stem cells (hDPSCs), and chondrocytes. Gene expression was compared between untreated controls (not differentiated) and cells subjected to chondrogenic differentiation. Data are presented as means ± *SD*. Statistical significance: * *p* < 0.05, ** *p* < 0.01, *** *p* < 0.001.

To assess chondrogenic potential, the expression levels of key cartilage-related genes (*Sox9*, *Col II*, *Acan*, *Col I*, and *Col X*) were measured in hAD-MSC, hDPSCs, and chondrocytes, cultured on PLCL under control conditions (not differentiated) or stimulated for chondrogenic differentiation. The results are presented as mean values (Figure 9). *Sox9*, a master transcription factor of chondrogenesis, was significantly upregulated upon chondrogenic induction in hAD-MSCs/PLCL (RQ 5.90, *p* < 0.05). hDPSCs/PLCL showed minimal *Sox9* induction, indicating limited activation of early chondrogenic pathways. Notably, under undifferentiated conditions, hDPSCs-PLCL exhibited a higher relative expression level of *Sox9* compared to untreated hAD-MSCs-PLCL (RQ 4.94 vs. 3.95) and even higher than native chondrocytes/PLCL (RQ 2.51) (Figure 9A). *Col II*, a marker of mature chondrocytes, was markedly upregulated in chondrocytes-PLCL (RQ 2.84). In the undifferentiated state, the highest *Col II* expression was observed in hAD-MSCs-PLCL, while hDPSCs-PLCL showed low expression levels when untreated (RQ 2.68 vs. 1.01). After chondrogenic induction, *Col II* expression remained relatively high in hAD-MSCs-PLCL (RQ 2.55), showing levels comparable to those observed prior to differentiation. hDPSCs-PLCL demonstrated an increase in expression following chondrogenic stimulation (RQ 2.20) (Figure 9B). The highest *Acan* expression level was observed in chondrocytes cultured on PLCL (RQ 2102.79). In hAD-MSCs/PLCL, *Acan* expression was substantially higher in the undifferentiated state than after chondrogenic induction, where it was markedly decreased (RQ 158.91 vs. 3.20, *p* < 0.01). In contrast, hDPSCs/PLCL exhibited low *Acan* expression under control conditions, but following chondrogenic stimulation, expression significantly increased, indicating enhanced matrix synthesis upon induction (RQ 4.94 vs. 279.28, *p* < 0.05) (Figure 9C). Chondrocytes cultured on PLCL exhibited very low *Col I* expression, representing the lowest level among all analyzed groups (RQ 0.84). In contrast, both hAD-MSCs/PLCL and hDPSCs/PLCL showed noticeably higher expression levels under undifferentiated conditions (RQ 2.00 and 1.38, *p* < 0.001) than chondrocytes/PLCL. Following chondrogenic induction, *Col I* expression further increased in both cell types, indicating substantial upregulation in response to differentiation stimuli (RQ 4.14 for hAD-MSCs/PLCL and 2.62 for hDPSCs/PLCL, *p* < 0.01) (Figure 9D). Chondrocytes cultured on PLCL showed moderate *Col II* expression level (RQ 5.89). hAD-MSCs-PLCL presented the highest expression in the control group (RQ 9.93), while hDPSCs-PLCL demonstrated low baseline levels when not differentiated (RQ 0.77). After chondrogenic induction, *Col X* expression decreased in hAD-MSCs-PLCL (RQ 0.27), whereas hDPSCs-PLCL exhibited a marked increase, indicating significant upregulation upon differentiation (RQ 5.89, *p* < 0.01) (Figure 9E).

## 4. Discussion

Therapy with autologous chondrocytes or BM-MSCs is often limited by poor survival of engraftment cells and uncontrolled chondrogenic differentiation of MSCs in vivo [2,65,66]. This limitation is not observed in tissue engineering, which is able to effectively create tissue that mimics native articular cartilage and restores joint function [29,30,34,40,46,52]. The objective of the present study was to assess the biological features and chondrogenic potential of hDPSCs and hAD-MSCs grown on PLCL by analyzing chondrogenic genes/proteins expression and comparing it with the phenotype of primary chondrocytes grown on a PLCL scaffold in order to design a bioconstruct for cartilage repair. Based on ISCT criteria, this study revealed that the hDPSCs and hAD-MSCs display typical MSC features and express molecules CD44, CD73, CD90, CD105, and STRO-1 at high levels and do not show the expression of the hematopoietic markers CD45 and HLA-DR [63]. Additionally, the mesenchymal/stromal cell features of hDPSCs and hAD-MSCs were also proven by their ability to differentiate in vitro into chondrocytes, osteoblasts, and adipocytes [48,67]. The obtained results showed that hDPSCs and hAD-MSCs displayed features recommended by the ISCT, which define their multipotent parameters and may be considered as a population of MSCs for use in tissue engineering [63,67]. Increasing evidence indicates that the chondrogenic potential of hDPSCs and hAD-MSCs grown on different synthetic scaffolds varies [35,36,67,68,69]. To our knowledge, there is no data comparing the expression of chondrogenic genes/proteins in chondrocytes derived from hDPSCs and hAD-MSCs upon chondrogenic induction on PLCL with the native phenotype of chondrocytes grown on PLCL. Based on our earlier data [48] showing that a hydrolytically modified PLCL nanofiber mat induces a suitable microenvironment for hDPSC growth and osteogenesis, thereby in the current study, we investigated the effect of the PLCL membrane on the chondrogenic potential of hDPSCs and hAD-MSCs and the biological behavior of analyzed stem/stromal cells and chondrocytes grown on PLCL. In agreement with a previous study, hDPSCs, hAD-MSCs, and chondrocytes cultured on the PLCL scaffold had similar metabolic activity rates to control cells at days 1 and 3, but higher for chondrocytes on PLCL and lower for hAD-MSCs on PLCL compared with control [48,70]. In line with several reports [48,51,53,70,71,72], we have confirmed that the PLCL scaffold is characterized by good biocompatibility because the number of cultured cells on PLCL increased from 1 to 7 days, which indicated that the chemical components of the PLCL scaffold promote hDPSC, hAD-MSC, and chondrocyte growth and proliferation. The nanofibrous architecture of PLCL promoted the attachment and distribution of hAD-MSCs, hDPSCs, and chondrocytes and did not induce changes in morphological features of stem/stromal cells and chondrocytes, which are in accordance with the previous reports [24,36,58]. We found that the adhesion, elongation, and distribution of stem/stromal cells and chondrocytes on a PLCL surface may depend on linear attachment points for cells on PLCL surface [53,54,70,73]. According to reports assessing the effect of PLCL, PLGA, and gelatin-PCL scaffolds on chondrocyte behavior, the good adhesion and high proliferation of chondrocytes grown on PLCL, observed in the current study, might reflect suitable biochemical interactions between the chondrocytes and PLCL composition [53,74,75,76]. Furthermore, our data indicated that PLCL provided a microenvironment that enhances hDPSC, hAD-MSC growth and promotes chondrocyte phenotypic stability, as has been shown by other authors [49,55,74,77]. Despite the promising results showing good biocompatibility of PLCL, it is worth emphasizing that the in vitro conditions do not reflect in vivo, so PLCL behavior might be different after implantation into damaged cartilage tissue [53]. In this study, F-actin in the stress fibers was found to be a key mediator of the chondrocyte phenotype, which is strongly involved in the generation of mechanical forces that drive the development chondrocytes was found in a majority of chondrocytes grown on PLCL after 12 days, and this observation was comparable with other studies [77,78]. The stabilization of F-actin stress fibers in chondrocytes is associated with actin polymerization, which has been shown to be an crucial regulator of the dedifferentiated chondrocyte phenotype acting in part via the actin-regulated transcription factor MRTF-A, leading to filamentous F-actin stress fiber arrangement, reducing cartilage matrix expression and their switch to a fibroblast-like phenotype and production of mechanically weak cartilage, which is in accordance with the published data [25,78,79]. This suggestion is supported by our results showing that culturing chondrocytes on PLCL over a period of 2 weeks not only produces GAG and expresses *Acan* and *Col II* markers of a cartilage/hyaline phenotype, but also expresses *Col I* and *Col X* markers of a hypertrophic phenotype, suggesting that the actin cytoskeleton status affected the chondrocyte phenotype [76,78,79]. This observation is consistent with other reports showing that bioengineered cartilage from a 3D culture of passaged chondrocytes in vitro leads to the reorganization of F-actin stress fibers into a cortical arrangement and promotes chondrocyte redifferentiation [80]. Several studies indicate that MSCs isolated from different tissues may exhibit biological diversity that can determine their chondrogenic potential and cell–scaffold interactions [24,35,81,82,83]. To verify this hypothesis, in the current study, the expression of chondrogenic-related genes/proteins and F-actin occurrence was analyzed in undifferentiated and differentiated hDPSCs and hAD-MSCs on PLCL and compared with native chondrocytes on PLCL. The lower expression of chondrogenic proteins in undifferentiated hDPSCs and hAD-MSCs on PLCL than in native chondrocytes on PLCL, observed in this study, indicates that the synthesis of chondrogenic proteins by hDPSCs and hAD-MSCs is associated with weak activation of chondrogenic genes [35,58,68,84]. Our results showed F-actin expression in the majority of hDPSCs and hAD-MSCs after 12 days of culture on PLCL, suggesting that the cells communicate with one another and cooperate with other microfilament-associated proteins and intercellular adhesion molecules (ICAMs), which influences the behavior of stem/stromal cells and determines their chondrogenic potential [80,84,85].

In the next step, we analyzed the chondrogenic potential of hDPSCs and hAD-MSCs grown on PLCL and compared chondrogenic genes/proteins expression and glycosaminoglycan (GAG) production in chondrocytes derived from differentiated stem/stromal cells on PLCL with the phenotype of primary chondrocytes cultured on PLCL. Consistent with many published reports showing the chondrogenic potential of hDPSCs and hAD-MSCs grown on different scaffolds, we noted that hDPSCs and hAD-MSCs upon chondrogenic differentiation on PLCL not only changed their stem/stromal phenotype into chondrogenic, but also changed their shape from fibroblastic to rounded polygonal [9,26,35,36,69,85,86]. Additionally, the chondrogenic capacity of hAD-MSCs and hDPSCs on PLCL was confirmed with Alcian Blue strong staining, suggesting the production of sulphated proteoglycans as an effective indicator of hDPSC and hAD-MSC chondrogenic differentiation, as was reported by other authors [35,67,87]. Interestingly, Alcian Blue staining of chondrocytes derived from hDPSCs and hAD-MSCs upon chondrogenic induction on the PLCL scaffold was comparable to the ECM production of primary chondrocytes on PLCL. These results are similar to other data showing that in hyaluronic acid and alginate scaffolds, GAG was produced, and *Col II* was expressed equally by AD-MSCs, BM-MSCs, and chondrocytes [88]. This observation suggests that hDPSCs and hAD-MSCs differentiate into functional chondrocytes on PLCL, inducing ECM deposition on the surface of the bioconstruct [35]. The chondrogenic potential of hDPSCs and hAD-MSCs grown on different scaffolds was investigated by measuring the proteins synthesis and genes expression of hyaline cartilage markers in differentiated stem/stromal cells [24,35,69,89]. The significantly higher expression of *Sox9*, *Col II*, and *Acan* proteins in differentiated hDPSCs and hAD-MSCs on PLCL compared to undifferentiated cells observed in the present study suggested a positive effect of the PLCL nanofiber scaffold on the chondrogenic potential of both stem/stromal cell types [9,24,36,69,85,86]. Similarly to the PLC, PLA, chitosan, and collagen scaffolds, our results indicated that the PLCL scaffold induced the chondrogenesis of hDPSCs and hAD-MSCs and that chondrocytes derived from differentiated hAD-MSCs and hDPSCs have a phenotype comparable to native chondrocytes [9,24,35,36,69,86]. The higher *Acan* protein expression found in chondrocytes derived from differentiated hAD-MSCs and hDPSCs on PLCL than in native chondrocytes and a comparable level *Col II* expression found in both groups of chondrocytes confirmed the chondrogenically differentiated status of the stem/stromal cells. These results are similar to studies showing that *Col II* and *Acan* are expressed in hDPSCs and hAD-MSCs exposed to a chondrogenic differentiation medium, and their chondrogenic potential may depend on the cell source, donor, and scaffold used as a platform for cells [24,35,67,68,69,76,83,87,88].

Moreover, according to some reports, the differences in *Col II* and *Acan* protein expression observed in this study in chondrocytes derived from hDPSCs/PLCL and hAD-MSCs/PLCL may depend on the activity of the *Sox9* transcription factor, which regulates *Col II* and *Acan* expression, and the activity of the *Acan* gene, which affects *Col II* expression [16,17,37]. Our results are in line with those obtained by Salvador-Clavell et al. [69], who detected a higher expression of *Col II* and *Acan* in chondrogenically induced hDPSCs than in control samples and suggested that microtissue enhances extracellular cartilage matrix production. Westin et al. [89] also observed that hDPSCs are able to effectively differentiate into chondrocytes on a porous chitosan–xanthan scaffold and form collagen fibers.

Similar to the results obtained by Ansar et al. [90] and Mardani et al. [88], we showed that the levels of *Col II* and *Acan* in differentiated hAD-MSCs on PLCL were comparable to those in native chondrocytes. Moreover, our results revealed that not only chondrocytes derived from hAD-MSCs and hDPSCs differentiated on PLCL demonstrated the expression of hypertrophic proteins, such as *Col I* and *Col X*, but is also found in native chondrocytes grown on PLCL at different levels. These results suggest that a hypertrophic chondrocyte population is not only generated during 28 days of hAD-MSC and hDPSC chondrogenesis, but also that this process occurs following 12 days of culture of chondrocytes on PLCL. According to published reports, there is a high possibility, as found in the current study, that hypertrophic chondrocytes start to secrete proangiogenic factors and metalloproteinases that enhance angiogenesis and matrix remodelling upon in vivo implantation [69,83,88]. Consequently, we assume that the cartilaginous tissue engineering using hAD-MSCs-PLCL and hDPSCs-PLCL bioconstruct could be eventually remodelled into bone by endochondral ossification pathway, increasing endochondral ossification pathway and matrix mineralization, leading to bone formation [91].

The phenotype of stem/stromal cell-derived chondrocytes and native chondrocytes on bioconstructs may depend on the expression of the *Sox9* gene, which inhibits the differentiation of chondrocytes into prohypertrophic chondrocytes and F-actin stress fibers expression, which promotes chondrocyte dedifferentiation and induces a hypertrophic phenotype [35,68,80,92]. The balance between expression *Sox9* and F-actin molecule might be crucial for chondrocyte phenotype [78,79].

Subsequently, an analysis of chondrogenic gene expression, such as *Sox9, Col II,* and *Acan* provided additional evidence on the chondrogenic potential of hDPSCs and hAD-MSCs grown on a PLCL scaffold. However, differentiated hAD-MSCs and hDPSCs on the PLCL scaffold displayed variable gene expression profiles compared to native chondrocytes on PLCL. The chondrogenically differentiated hAD-MSCs, hDPSCs, and primary chondrocytes on PLCL had comparable expression of *Col II* but different expression of *Acan* and *Sox9* genes. This was reflected in the ECM staining, which showed a similar uptake profile. Lee at al. [68] also observed such differences and suggested that *Sox9* plays a key role in cartilage formation in the early stage of chondrogenesis and regulates *Col II* and *Acan* expression. The findings of this study align with other studies showing that the chondrogenic potential of hAD-MSCs and hDPSCs grown on synthetic scaffolds is associated with the scaffold geometry, which might have a positive influence on the synthesis of cartilage-specific molecules in differentiated cells [9,24,35,36,69,85]. On the other hand, a comparable *Col II* expression in native chondrocytes and those derived from hAD-MSCs and hDPSCs, but higher *Acan* expression in native chondrocytes seeded on PLCL, indicated the bioactive ability of the scaffold to support chondrogenic differentiation and stabilize the phenotype of hyaline cartilage chondrocytes [24]. We observed that chondrogenically differentiated hAD-MSCs and hDPSCs on PLCL displayed a significantly higher level of *Col I* than undifferentiated stem/stromal cells and chondrocytes. These results suggested early phenotypic drift in vitro that induced fibrocartilage-like matrix production under chondrogenic conditions [36,85]. The high expression of *Col X* mRNA found in native chondrocytes/PLCL and hDPSCs/PLCL after chondrogenic induction in vitro indicated that among native chondrocytes and differentiated hDPSCs, there are chondrocytes with hypertrophic phenotype, which limits their clinical application in cartilage tissue regeneration because native hypertrophic chondrocytes or hypertrophic differentiation of hDPSCs-derived chondrocytes can mediate crosstalk by regulating cell-matrix degradation and osteoclast recruitment and transdifferentiating into osteoprogenitors and mature osteoblasts, causing bone matrix synthesis [35,67]. Transformation into hypertrophic chondrocytes remains an important matter that needs to be further elucidated to improve the regenerative potential of stem/stromal cells in cartilage repair. As demonstrated in a previous study showing the usefulness of PLCL in bone regeneration, our results showed that the PLCL membrane is a suitable platform for stem/stromal cells and chondrocytes and may be used in cartilage tissue regeneration [53,60,74,93]. However, there are some limitations: firstly, the architecture of PLCL scaffold is not similar to that of cartilage tissue; secondly, selecting appropriate cell sources for cartilage tissue engineering, whose quality and quantity are donor-dependent; thirdly, there are no in vivo experiments that are needed to show the regenerative potential of hDPSC- and hAD-MSCs-PLCL and chondrocytes-PLCL bioimplants.

## 5. Conclusions

The results of this study provide solid evidence that the PLCL scaffold promotes the chondrogenic differentiation of hAD-MSCs and hDPSCs toward chondrocytes with phenotypic similarities to native chondrocytes. Moreover, the results highlighted that the PLCL scaffold composition has a positive effect on the behavior of hDPSCs, hAD-MSCs, and chondrocytes and that the cell–PLCL interaction may affect chondrogenic gene expression and matrix protein synthesis. However, further research is needed to recommend hAD-MSCs-PLCL and hDPSCs-PLCL bioconstructs for cartilage tissue engineering.

## Figures and Tables

**Table 1:** Primers used in qPCR.

Gene	Full Name	Primer Sequences (5′-3′)
*B2M*	*β-2 microglobulin*	F: AATGCGGCATCTTCAAACCT R: TGACTTTGTCACAGCCCAAGATA
*Sox9*	*SRY-box transcription factor 9*	F: TGGGCAAGCTCTGGAGACTTC R: ATCCGGGTGGTCCTTCTTGTG
*Col II*	*Collagen type II*	F: CGTCCAGATGACCTTCCTACG R: TGAGCAGGGCCTTCTTGAG
*Acan*	*Aggrecan*	F: AGGCAGCGTGATCCTTACC R: GCCCTCTCCAGTCTCATTCTC
*Col I*	*Collagen type I*	F: AGGTGCTGATGGCTCTCCT R: TGTTCCCACTTTCACCAGG
*Col X*	*Collagen type X*	F: GCAACTAAGGGCCTCAATGG R: CTCAGGCATGACTGCTTGAC

## Data Availability

The original contributions presented in this study are included in the article. Further inquiries can be directed to the corresponding author.

## Supplementary Materials

The following supporting information can be downloaded at: https://www.mdpi.com/article/10.3390/cells15131168/s1, Figure S1. Morphological and phenotypic characterization of hDPSCs and hAD-MSCs cultured as a monolayer. Representative image of (A) hDPSCs and (B) hAD-MSCs after 12 d of culturing examined under an inverted light microscope, showing good adhesion and fibroblastic morphology. Representative flow cytometry analysis of hDPSC and hAD-MSC phenotypes. (C) hDPSCs and (D) hAD-MSCs were positive for naïve MSCs biomarkers CD73+/CD90+/CD105+/CD44 and Stro-1, positive for HLA ABC antigen, and negative for HLA DR and CD31, CD45 expression. Red-filled histograms correspond to MSCs labelled with the analyzed antibodies, and empty histograms illustrate isotype controls. For Figure S1A,B scale bars: 100 µm. Figure S2. Lineage differentiation potential of hDPSCs and hAD-MSCs. (A,B) Osteogenesis was detected by Alizarin Red S staining of mineralized nodules. (C,D) Chondrogenesis was demonstrated by Alcian Blue matrix staining. (E,F) Adipogenesis was detected by Oil Red O staining of lipid droplet formation. Negative control cells cultured in non-differentiation α-MEM medium showed as stars* in square. In panel (A–D,F), scale bars: 100 µm and (E) 50 µm.

## Abbreviations

The following abbreviations are used in this manuscript:

hAD-MSCs	Human adipose-derived stem cells
hDPSCs	Human dental pulp stem cells
PLCL	Poly(L-lactide-*co*-caprolactone)
RT-PCR	Real-time polymerase chain reaction
*Col II*	*Collagen type II*
*Acan*	*Aggrecan*
OA	Osteoarthritis
BM-MSCs	Bone marrow mesenchymal stem cells
ACI	Autologous chondrocyte implantation
MACI	Matrix-assisted autologous chondrocyte implantation
MSCs	Mesenchymal stem cells
SVF	Stromal vascular fraction
MFAT	Micro-fragmented adipose tissue
ECM	Extracellular matrix
MMP-13	Matrix metaloproteinase-13
PGA	Polyglycol acid
GPC	Gel permeation chromatography
SEM	Scanning electron microscopy
α-MEM	α-minimum essential medium
ISCT	International Society for Cellular
CD	Cluster differentiation
DMEM/F12	Dulbecco’s Modified Eagle Medium/Nutrient Mixture F12
*SD*	Standard deviation
H&E	Hematoxylin and eosin
RT	Room temperature
PBS	Phosphate-buffered saline
DAPI	4′,6-diamidino-2-phenylindole
GAG	Sulfated glycosaminoglycans
*Col I*	*Collagen type I*
*Col X*	*Collagen type X*
TBS	Tris-buffered saline
DAB	3,3 diaminobenzidine
RNA	Ribonucleic acid
RNeasy	Ribonuclease
cDNA	Complementary DNA
*B2M*	*β-2 microglobulin*
MTT	Mean transit time
RQ	Relative quantity
HLA-DR	Human Leukocyte Antigen–DR isotype
Gelatin-PCL	Gelatin-polycaprolactone
ICAMs	Intercellular adhesion molecules

## References

[1] Owaidah A.Y. (2024). Induced Pluripotent Stem Cells in Cartilage Tissue Engineering: A Literature Review. Biosci. Rep..

[2] Zelinka A., Roelofs A.J., Kandel R.A., De Bari C. (2022). Cellular Therapy and Tissue Engineering for Cartilage Repair. Osteoarthr. Cartil..

[3] Marchan J., Wittig O., Diaz-Solano D., Gomez M., Cardier J.E. (2022). Enhanced Chondrogenesis from Chondrocytes Co-Cultured on Mesenchymal Stromal Cells: Implication for Cartilage Repair. Injury.

[4] Wang P., Zhang S., Meng Q., Zhu P., Yuan W. (2022). Treatment and Application of Stem Cells from Different Sources for Cartilage Injury: A Literature Review. Ann. Transl. Med..

[5] Grevenstein D., Mamilos A., Schmitt V.H., Niedermair T., Wagner W., Kirkpatrick C.J., Brochhausen C. (2021). Excellent Histological Results in Terms of Articular Cartilage Regeneration after Spheroid-Based Autologous Chondrocyte Implantation (ACI). Knee Surg. Sports Traumatol. Arthrosc..

[6] Si Z., Wang X., Sun C., Kang Y., Xu J., Wang X., Hui Y. (2019). Adipose-Derived Stem Cells: Sources, Potency, and Implications for Regenerative Therapies. Biomed. Pharmacother..

[7] Rodeo S.A. (2019). Cell Therapy in Orthopaedics: Where Are We in 2019?. Bone Jt. J..

[8] Puiggalí-Jou A., Asadikorayem M., Maniura-Weber K., Zenobi-Wong M. (2023). Growth Factor–Loaded Sulfated Microislands in Granular Hydrogels Promote hMSCs Migration and Chondrogenic Differentiation. Acta Biomater..

[9] Song C., Wu X., Wei Z., Xu Y., Wang Y., Zhao Y. (2024). Dental Pulp Stem Cells-Loaded Kartogenin-Modified Hydrogel Microspheres with Chondrocyte Differentiation Property for Cartilage Repair. Chem. Eng. J..

[10] Gomez M., Wittig O., Diaz-Solano D., Cardier J.E. (2021). Mesenchymal Stromal Cell Transplantation Induces Regeneration of Large and Full-Thickness Cartilage Defect of the Temporomandibular Joint. Cartilage.

[11] Hernigou P., Delambre J., Quiennec S., Poignard A. (2021). Human Bone Marrow Mesenchymal Stem Cell Injection in Subchondral Lesions of Knee Osteoarthritis: A Prospective Randomized Study versus Contralateral Arthroplasty at a Mean Fifteen Year Follow-Up. Int. Orthop..

[12] Le H., Xu W., Zhuang X., Chang F., Wang Y., Ding J. (2020). Mesenchymal Stem Cells for Cartilage Regeneration. J. Tissue Eng..

[13] Brose T.Z., Kubosch E.J., Schmal H., Stoddart M.J., Armiento A.R. (2021). Crosstalk Between Mesenchymal Stromal Cells and Chondrocytes: The Hidden Therapeutic Potential for Cartilage Regeneration. Stem Cell Rev. Rep..

[14] Jiang S., Tian G., Li X., Yang Z., Wang F., Tian Z., Huang B., Wei F., Zha K., Sun Z. (2021). Research Progress on Stem Cell Therapies for Articular Cartilage Regeneration. Stem Cells Int..

[15] Lv Z., Cai X., Bian Y., Wei Z., Zhu W., Zhao X., Weng X. (2023). Advances in Mesenchymal Stem Cell Therapy for Osteoarthritis: From Preclinical and Clinical Perspectives. Bioengineering.

[16] Epanomeritakis I.E., Lee E., Lu V., Khan W. (2022). The Use of Autologous Chondrocyte and Mesenchymal Stem Cell Implants for the Treatment of Focal Chondral Defects in Human Knee Joints-A Systematic Review and Meta-Analysis. Int. J. Mol. Sci..

[17] Sun Q., Zhuang Z., Bai R., Deng J., Xin T., Zhang Y., Li Q., Han B. (2023). Lysine 68 Methylation-Dependent SOX9 Stability Control Modulates Chondrogenic Differentiation in Dental Pulp Stem Cells. Adv. Sci..

[18] Garcia-Ruiz A., Sánchez-Domínguez C.N., Moncada-Saucedo N.K., Pérez-Silos V., Lara-Arias J., Marino-Martínez I.A., Camacho-Morales A., Romero-Diaz V.J., Peña-Martinez V., Ramos-Payán R. (2021). Sequential Growth Factor Exposure of Human Ad-MSCs Improves Chondrogenic Differentiation in an Osteochondral Biphasic Implant. Exp. Ther. Med..

[19] Shen W.-C., Lai Y.-C., Li L.-H., Liao K., Lai H.-C., Kao S.-Y., Wang J., Chuong C.-M., Hung S.-C. (2019). Methylation and PTEN Activation in Dental Pulp Mesenchymal Stem Cells Promotes Osteogenesis and Reduces Oncogenesis. Nat. Commun..

[20] Xiang X.-N., Zhu S.-Y., He H.-C., Yu X., Xu Y., He C.-Q. (2022). Mesenchymal Stromal Cell-Based Therapy for Cartilage Regeneration in Knee Osteoarthritis. Stem Cell Res. Ther..

[21] Epanomeritakis I.E., Khan W.S. (2024). Adipose-Derived Regenerative Therapies for the Treatment of Knee Osteoarthritis. World J. Stem Cells.

[22] Jeyaraman M., Muthu S., Ganie P.A. (2021). Does the Source of Mesenchymal Stem Cell Have an Effect in the Management of Osteoarthritis of the Knee? Meta-Analysis of Randomized Controlled Trials. Cartilage.

[23] Goncharov E.N., Koval O.A., Bezuglov E.N., Ramirez M.d.J.E., Engelgard M., Igorevich E.I., Saporiti A., Kotenko K.V., Montemurro N. (2023). Stromal Vascular Fraction Therapy for Knee Osteoarthritis: A Systematic Review. Medicina.

[24] Eldeen G.N., Elkhooly T.A., El Bassyouni G.T., Hamdy T.M., Hawash A.R., Aly R.M. (2024). Enhancement of the Chondrogenic Differentiation Capacity of Human Dental Pulp Stem Cells via Chondroitin Sulfate-Coated Polycaprolactone-MWCNT Nanofibers. Sci. Rep..

[25] Lee W.-S., Kim H.J., Kim K.-I., Kim G.B., Jin W. (2019). Intra-Articular Injection of Autologous Adipose Tissue-Derived Mesenchymal Stem Cells for the Treatment of Knee Osteoarthritis: A Phase IIb, Randomized, Placebo-Controlled Clinical Trial. Stem Cells Transl. Med..

[26] Yokota N., Lyman S., Hanai H., Shimomura K., Ando W., Nakamura N. (2022). Clinical Safety and Effectiveness of Adipose-Derived Stromal Cell vs Stromal Vascular Fraction Injection for Treatment of Knee Osteoarthritis: 2-Year Results of Parallel Single-Arm Trials. Am. J. Sports Med..

[27] Gomez-Salazar M., Gonzalez-Galofre Z.N., Casamitjana J., Crisan M., James A.W., Péault B. (2020). Five Decades Later, Are Mesenchymal Stem Cells Still Relevant?. Front. Bioeng. Biotechnol..

[28] Barry F. (2019). MSC Therapy for Osteoarthritis: An Unfinished Story. J. Orthop. Res..

[29] Wasyłeczko M., Sikorska W., Chwojnowski A. (2020). Review of Synthetic and Hybrid Scaffolds in Cartilage Tissue Engineering. Membranes.

[30] Yang C., Chen R., Chen C., Yang F., Xiao H., Geng B., Xia Y. (2024). Tissue Engineering Strategies Hold Promise for the Repair of Articular Cartilage Injury. Biomed. Eng. Online.

[31] Krishani M., Shin W.Y., Suhaimi H., Sambudi N.S. (2023). Development of Scaffolds from Bio-Based Natural Materials for Tissue Regeneration Applications: A Review. Gels.

[32] Uzieliene I., Bironaite D., Bernotas P., Sobolev A., Bernotiene E. (2021). Mechanotransducive Biomimetic Systems for Chondrogenic Differentiation In Vitro. Int. J. Mol. Sci..

[33] Wang H., Zhang J., Liu H., Wang Z., Li G., Liu Q., Wang C. (2023). Chondrocyte-Laden Gelatin/Sodium Alginate Hydrogel Integrating 3D Printed PU Scaffold for Auricular Cartilage Reconstruction. Int. J. Biol. Macromol..

[34] Thorp H., Kim K., Kondo M., Maak T., Grainger D.W., Okano T. (2021). Trends in Articular Cartilage Tissue Engineering: 3D Mesenchymal Stem Cell Sheets as Candidates for Engineered Hyaline-Like Cartilage. Cells.

[35] Piperigkou Z., Bainantzou D., Makri N., Papachristou E., Mantsou A., Choli-Papadopoulou T., Theocharis A.D., Karamanos N.K. (2023). Enhancement of Mesenchymal Stem Cells’ Chondrogenic Potential by Type II Collagen-Based Bioscaffolds. Mol. Biol. Rep..

[36] Lin I.-C., Wang T.-J., Wu C.-L., Lu D.-H., Chen Y.-R., Yang K.-C. (2020). Chitosan-Cartilage Extracellular Matrix Hybrid Scaffold Induces Chondrogenic Differentiation to Adipose-Derived Stem Cells. Regen. Ther..

[37] Gao Q., Xie C., Wang P., Xie M., Li H., Sun A., Fu J., He Y. (2020). 3D Printed Multi-Scale Scaffolds with Ultrafine Fibers for Providing Excellent Biocompatibility. Mater. Sci. Eng. C Mater. Biol. Appl..

[38] Beltran F.O., Arabiyat A.S., Culibrk R.A., Yeisley D.J., Houk C.J., Hicks A.J., Negrón Hernández J., Nitschke B.M., Hahn M.S., Grunlan M.A. (2023). Enhanced Degradation and Bioactivity in Polysiloxane-Based Shape Memory Polymer (SMP) Scaffolds. Polymer.

[39] Janoušková O. (2018). Synthetic Polymer Scaffolds for Soft Tissue Engineering. Physiol. Res..

[40] Kalkan R., Nwekwo C.W., Adali T. (2018). The Use of Scaffolds in Cartilage Regeneration. Crit. Rev. Eukaryot. Gene Expr..

[41] Marycz K., Smieszek A., Targonska S., Walsh S.A., Szustakiewicz K., Wiglusz R.J. (2020). Three Dimensional (3D) Printed Polylactic Acid with Nano-Hydroxyapatite Doped with Europium(III) Ions (nHAp/PLLA@Eu3+) Composite for Osteochondral Defect Regeneration and Theranostics. Mater. Sci. Eng. C.

[42] Venkatesan J.K., Falentin-Daudré C., Leroux A., Migonney V., Cucchiarini M. (2020). Biomaterial-Guided Recombinant Adeno-Associated Virus Delivery from Poly(Sodium Styrene Sulfonate)-Grafted Poly(ɛ-Caprolactone) Films to Target Human Bone Marrow Aspirates. Tissue Eng. Part A.

[43] Lam A.T.L., Reuveny S., Oh S.K.-W. (2020). Human Mesenchymal Stem Cell Therapy for Cartilage Repair: Review on Isolation, Expansion, and Constructs. Stem Cell Res..

[44] Khan A.R., Gholap A.D., Grewal N.S., Jun Z., Khalid M., Zhang H.-J. (2025). Advances in Smart Hybrid Scaffolds: A Strategic Approach for Regenerative Clinical Applications. Eng. Regen..

[45] Maihemuti A., Zhang H., Lin X., Wang Y., Xu Z., Zhang D., Jiang Q. (2023). 3D-Printed Fish Gelatin Scaffolds for Cartilage Tissue Engineering. Bioact. Mater..

[46] Setayeshmehr M., Esfandiari E., Rafieinia M., Hashemibeni B., Taheri-Kafrani A., Samadikuchaksaraei A., Kaplan D.L., Moroni L., Joghataei M.T. (2019). Hybrid and Composite Scaffolds Based on Extracellular Matrices for Cartilage Tissue Engineering. Tissue Eng. Part B Rev..

[47] Setayeshmehr M., Esfandiari E., Hashemibeni B., Tavakoli A.H., Rafienia M., Samadikuchaksaraei A., Moroni L., Joghataei M.T. (2019). Chondrogenesis of Human Adipose-Derived Mesenchymal Stromal Cells on the [Devitalized Costal Cartilage Matrix/Poly(Vinyl Alcohol)/Fibrin] Hybrid Scaffolds. Eur. Polym. J..

[48] Bar J.K., Lis-Nawara A., Kowalczyk T., Grelewski P.G., Stamnitz S., Gerber H., Klimczak A. (2023). Osteogenic Potential of Human Dental Pulp Stem Cells (hDPSCs) Growing on Poly L-Lactide-Co-Caprolactone and Hyaluronic Acid (HYAFF-11TM) Scaffolds. Int. J. Mol. Sci..

[49] Bistolfi A., Ferracini R., Galletta C., Tosto F., Sgarminato V., Digo E., Vernè E., Massè A. (2017). Regeneration of Articular Cartilage: Scaffold Used in Orthopedic Surgery. A short handbook of available products for regenerative joints surgery. Clin. Sci. Res. Rep..

[50] He Y., Liu W., Guan L., Chen J., Duan L., Jia Z., Huang J., Li W., Liu J., Xiong J. (2018). A 3D-Printed PLCL Scaffold Coated with Collagen Type I and Its Biocompatibility. Biomed. Res. Int..

[51] Jia Z., Li H., Cao R., Xiao K., Lu J., Zhao D., Wang Z., Zhang Y., Chen J., Zhang W. (2020). Electrospun Nanofibrous Membrane of Fish Collagen/Polycaprolactone for Cartilage Regeneration. Am. J. Transl. Res..

[52] Baranowski M., Wasyłeczko M., Kosowska A., Plichta A., Kowalczyk S., Chwojnowski A., Bielecki W., Czubak J. (2022). Regeneration of Articular Cartilage Using Membranes of Polyester Scaffolds in a Rabbit Model. Pharmaceutics.

[53] Chen Y., Xu W., Shafiq M., Tang J., Hao J., Xie X., Yuan Z., Xiao X., Liu Y., Mo X. (2021). Three-Dimensional Porous Gas-Foamed Electrospun Nanofiber Scaffold for Cartilage Regeneration. J. Colloid. Interface Sci..

[54] Pacilio S., Costa R., Papa V., Rodia M.T., Gotti C., Pagnotta G., Cenacchi G., Focarete M.L. (2023). Electrospun Poly(L-Lactide-Co-ε-Caprolactone) Scaffold Potentiates C2C12 Myoblast Bioactivity and Acts as a Stimulus for Cell Commitment in Skeletal Muscle Myogenesis. Bioengineering.

[55] Reddy V.S., Tian Y., Zhang C., Ye Z., Roy K., Chinnappan A., Ramakrishna S., Liu W., Ghosh R. (2021). A Review on Electrospun Nanofibers Based Advanced Applications: From Health Care to Energy Devices. Polymers.

[56] Qiao K., Xu L., Tang J., Wang Q., Lim K.S., Hooper G., Woodfield T.B.F., Liu G., Tian K., Zhang W. (2022). The Advances in Nanomedicine for Bone and Cartilage Repair. J. Nanobiotechnol..

[57] Jakobsen R.B., Shahdadfar A., Reinholt F.P., Brinchmann J.E. (2010). Chondrogenesis in a Hyaluronic Acid Scaffold: Comparison between Chondrocytes and MSC from Bone Marrow and Adipose Tissue. Knee Surg. Sports Traumatol. Arthrosc..

[58] Mahmoudifar N., Doran P.M. (2010). Extent of Cell Differentiation and Capacity for Cartilage Synthesis in Human Adult Adipose-Derived Stem Cells: Comparison with Fetal Chondrocytes. Biotechnol. Bioeng..

[59] Seda Tigli R., Ghosh S., Laha M.M., Shevde N.K., Daheron L., Gimble J., Gümüşderelioglu M., Kaplan D.L. (2009). Comparative Chondrogenesis of Human Cell Sources in 3D Scaffolds. J. Tissue Eng. Regen. Med..

[60] Bar J.K., Kowalczyk T., Grelewski P.G., Stamnitz S., Paprocka M., Lis J., Lis-Nawara A., An S., Klimczak A. (2022). Characterization of Biological Properties of Dental Pulp Stem Cells Grown on an Electrospun Poly(l-Lactide-Co-Caprolactone) Scaffold. Materials.

[61] Deszcz I., Lis-Nawara A., Grelewski P., Dragan S., Bar J. (2020). Utility of Direct 3D Co-Culture Model for Chondrogenic Differentiation of Mesenchymal Stem Cells on Hyaluronan Scaffold (Hyaff-11). Regen. Biomater..

[62] Kozlowska U., Krawczenko A., Futoma K., Jurek T., Rorat M., Patrzalek D., Klimczak A. (2019). Similarities and Differences between Mesenchymal Stem/Progenitor Cells Derived from Various Human Tissues. World J. Stem Cells.

[63] Viswanathan S., Shi Y., Galipeau J., Krampera M., Leblanc K., Martin I., Nolta J., Phinney D.G., Sensebe L. (2019). Mesenchymal Stem versus Stromal Cells: International Society for Cell & Gene Therapy (ISCT^®^) Mesenchymal Stromal Cell Committee Position Statement on Nomenclature. Cytotherapy.

[64] Heidari M., Salehkhou S., Heidari-Vala H., Akhondi M.M. (2011). In Vitro Human Chondrocyte Culture; A Modified Protocol. Middle-East J. Sci. Res..

[65] Mahdavi-Jouibari F., Parseh B., Kazeminejad E., Khosravi A. (2023). Hopes and Opportunities of Stem Cells from Human Exfoliated Deciduous Teeth (SHED) in Cartilage Tissue Regeneration. Front. Bioeng. Biotechnol..

[66] Kim K., Bou-Ghannam S., Kameishi S., Oka M., Grainger D.W., Okano T. (2021). Allogeneic Mesenchymal Stem Cell Sheet Therapy: A New Frontier in Drug Delivery Systems. J. Control Release.

[67] Labedz-Maslowska A., Bryniarska N., Kubiak A., Kaczmarzyk T., Sekula-Stryjewska M., Noga S., Boruczkowski D., Madeja Z., Zuba-Surma E. (2020). Multilineage Differentiation Potential of Human Dental Pulp Stem Cells-Impact of 3D and Hypoxic Environment on Osteogenesis In Vitro. Int. J. Mol. Sci..

[68] Lee J., Lee C.Y., Park J.-H., Seo H.-H., Shin S., Song B.-W., Kim I.-K., Kim S.W., Lee S., Park J.-C. (2020). Differentiation of Adipose-Derived Stem Cells into Functional Chondrocytes by a Small Molecule That Induces Sox9. Exp. Mol. Med..

[69] Salvador-Clavell R., Martín de Llano J.J., Milián L., Oliver M., Mata M., Carda C., Sancho-Tello M. (2021). Chondrogenic Potential of Human Dental Pulp Stem Cells Cultured as Microtissues. Stem Cells Int..

[70] Wang Z., Lin M., Xie Q., Sun H., Huang Y., Zhang D., Yu Z., Bi X., Chen J., Wang J. (2016). Electrospun Silk Fibroin/Poly(Lactide-Co-ε-Caprolactone) Nanofibrous Scaffolds for Bone Regeneration. Int. J. Nanomed..

[71] de Souza J.R., Cardoso L.M., de Toledo P.T.A., Rahimnejad M., Kito L.T., Thim G.P., Campos T.M.B., Borges A.L.S., Bottino M.C. (2024). Biodegradable Electrospun Poly(L-Lactide-Co-ε-Caprolactone)/Polyethylene Glycol/Bioactive Glass Composite Scaffold for Bone Tissue Engineering. J. Biomed. Mater. Res. B Appl. Biomater..

[72] Rozila I., Azari P., Munirah S., Safwani W.K.Z.W., Pingguan-Murphy B., Chua K.H. (2021). Polycaprolactone-Based Scaffolds Facilitates Osteogenic Differentiation of Human Adipose-Derived Stem Cells in a Co-Culture System. Polymers.

[73] Alipour M., Aghazadeh M., Akbarzadeh A., Vafajoo Z., Aghazadeh Z., Raeisdasteh Hokmabad V. (2019). Towards Osteogenic Differentiation of Human Dental Pulp Stem Cells on PCL-PEG-PCL/Zeolite Nanofibrous Scaffolds. Artif. Cells Nanomed. Biotechnol..

[74] Chen Y., Jia Z., Shafiq M., Xie X., Xiao X., Castro R., Rodrigues J., Wu J., Zhou G., Mo X. (2021). Gas Foaming of Electrospun Poly(L-Lactide-Co-Caprolactone)/Silk Fibroin Nanofiber Scaffolds to Promote Cellular Infiltration and Tissue Regeneration. Colloids Surf. B Biointerfaces.

[75] Chen W., Xu Y., Liu Y., Wang Z., Li Y., Jiang G., Mo X., Zhou G. (2019). Three-Dimensional Printed Electrospun Fiber-Based Scaffold for Cartilage Regeneration. Mater. Des..

[76] Li Y., Liu Y., Xun X., Zhang W., Xu Y., Gu D. (2019). Three-Dimensional Porous Scaffolds with Biomimetic Microarchitecture and Bioactivity for Cartilage Tissue Engineering. ACS Appl. Mater. Interfaces.

[77] Yang P., Xiao Y., Chen L., Yang C., Cheng Q., Li H., Chen D., Wu J., Liao Z., Yang C. (2024). Targeting Fascin1 Maintains Chondrocytes Phenotype and Attenuates Osteoarthritis Development. Bone Res..

[78] Delve E., Co V., Kandel R.A. (2020). Superficial and Deep Zone Articular Chondrocytes Exhibit Differences in Actin Polymerization Status and Actin-Associated Molecules in Vitro. Osteoarthr. Cart. Open.

[79] Schofield M.M., Rzepski A.T., Richardson-Solorzano S., Hammerstedt J., Shah S., Mirack C.E., Herrick M., Parreno J. (2024). Targeting F-Actin Stress Fibers to Suppress the Dedifferentiated Phenotype in Chondrocytes. Eur. J. Cell Biol..

[80] Davis E.E.R., Manzoni T.J., Bianchi V.J., Weber J.F., Wu P.H., Regmi S.C., Waldman S.D., Schmidt T.A., Su A.W., Kandel R.A. (2024). Passaged Articular Chondrocytes From the Superficial Zone and Deep Zone Can Regain Zone-Specific Properties After Redifferentiation. Am. J. Sports Med..

[81] Wuttisiriboon K., Tippayawat P., Daduang J., Limpaiboon T. (2023). Three-Dimensional Silk Fibroin-Gelatin/Chondroitin Sulfate/Hyaluronic Acid-Aloe Vera Scaffold Supports in Vitro Chondrogenesis of Bone Marrow Mesenchymal Stem Cells and Reduces Inflammatory Effect. J. Biomed. Mater. Res. Part B Appl. Biomater..

[82] Vasiliadis A.V., Galanis N. (2020). Effectiveness of AD-MSCs Injections for the Treatment of Knee Osteoarthritis: Analysis of the Current Literature. J. Stem Cells Regen. Med..

[83] Chijimatsu R., Miwa S., Okamura G., Miyahara J., Tachibana N., Ishikura H., Higuchi J., Maenohara Y., Tsuji S., Sameshima S. (2021). Divergence in Chondrogenic Potential between in Vitro and in Vivo of Adipose- and Synovial-Stem Cells from Mouse and Human. Stem Cell Res. Ther..

[84] Fan Y.-L., Zhao H.-C., Li B., Zhao Z.-L., Feng X.-Q. (2019). Mechanical Roles of F-Actin in the Differentiation of Stem Cells: A Review. ACS Biomater. Sci. Eng..

[85] Yang K.-C., Chen I.-H., Yang Y.-T., Hsiao J.-K., Wang C.-C. (2020). Effects of Scaffold Geometry on Chondrogenic Differentiation of Adipose-Derived Stem Cells. Mater. Sci. Eng. C Mater. Biol. Appl..

[86] Amsar R.M., Barlian A., Judawisastra H., Wibowo U.A., Karina K. (2021). Cell Penetration and Chondrogenic Differentiation of Human Adipose Derived Stem Cells on 3D Scaffold. Future Sci. OA.

[87] Mohamed-Ahmed S., Fristad I., Lie S.A., Suliman S., Mustafa K., Vindenes H., Idris S.B. (2018). Adipose-Derived and Bone Marrow Mesenchymal Stem Cells: A Donor-Matched Comparison. Stem Cell Res. Ther..

[88] Mardani M., Hashemibeni B., Ansar M.M., Zarkesh Esfahani S.H., Kazemi M., Goharian V., Esmaeili N., Esfandiary E. (2013). Comparison between Chondrogenic Markers of Differentiated Chondrocytes from Adipose Derived Stem Cells and Articular Chondrocytes In Vitro. Iran. J. Basic. Med. Sci..

[89] Westin C.B., Trinca R.B., Zuliani C., Coimbra I.B., Moraes Â.M. (2017). Differentiation of Dental Pulp Stem Cells into Chondrocytes upon Culture on Porous Chitosan-Xanthan Scaffolds in the Presence of Kartogenin. Mater. Sci. Eng. C Mater. Biol. Appl..

[90] Ansar M.M., Esfandiariy E., Mardani M., Hashemibeni B., Zarkesh-Esfahani S.H., Hatef M., Kabiri A. (2012). A Comparative Study of Aggrecan Synthesis between Natural Articular Chondrocytes and Differentiated Chondrocytes from Adipose Derived Stem Cells in 3D Culture. Adv. Biomed. Res..

[91] Suresh A., Parasuraman G., Priya M., Rebekah G., Jeyaraj C., Vinod E. (2025). Evaluation of Chondrogenesis and Osteogenesis in Human Mesenchymal Stem Cells, Chondrocytes, and Chondroprogenitors Using Molecular Markers, Cellular Markers and Polarized Microscopy. Differentiation.

[92] Bianchi V.J., Lee A., Anderson J., Parreno J., Theodoropoulos J., Backstein D., Kandel R. (2019). Redifferentiated Chondrocytes in Fibrin Gel for the Repair of Articular Cartilage Lesions. Am. J. Sports Med..

[93] Šebová E., Leal F., Klusáček Rampichová M., Nirwan V.P., Fahmi A., Costa P.F., Filová E. (2025). Electrospun Poly(L-Lactide-Co-ε-Caprolactone) Nanofibers with Hydroxyapatite Nanoparticles Mimic Cellular Interplay in Bone Regeneration. Int. J. Mol. Sci..

