# Supplementary figures and images for: Chondrogenic Potential of Human Adipose-Derived Stem/Stromal Cells (hAD-MSCs) and Human Dental Pulp Stem/Stromal Cells (hDPSCs) Growing on a Poly L-Lactide-Co-Caprolactone Scaffold (PLCL)
Source: Cells. 2026 Jun 26;15(13):1168. doi: 10.3390/cells15131168 (PMC13359943; doi:10.3390/cells15131168)

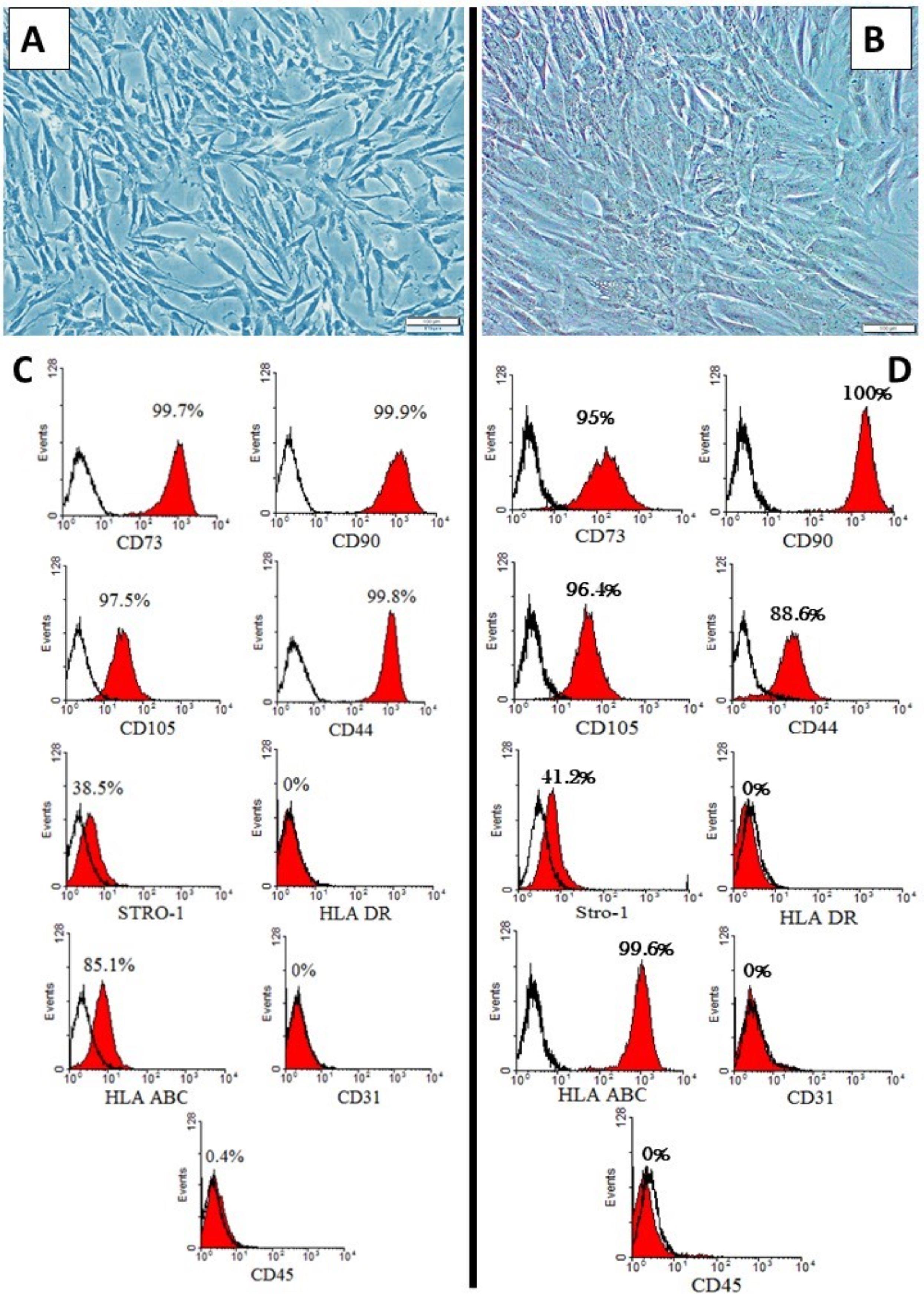

Supplement: Supplementary file 1 [file cells-15-01168-s001.zip › Suppl. 1.jpg]

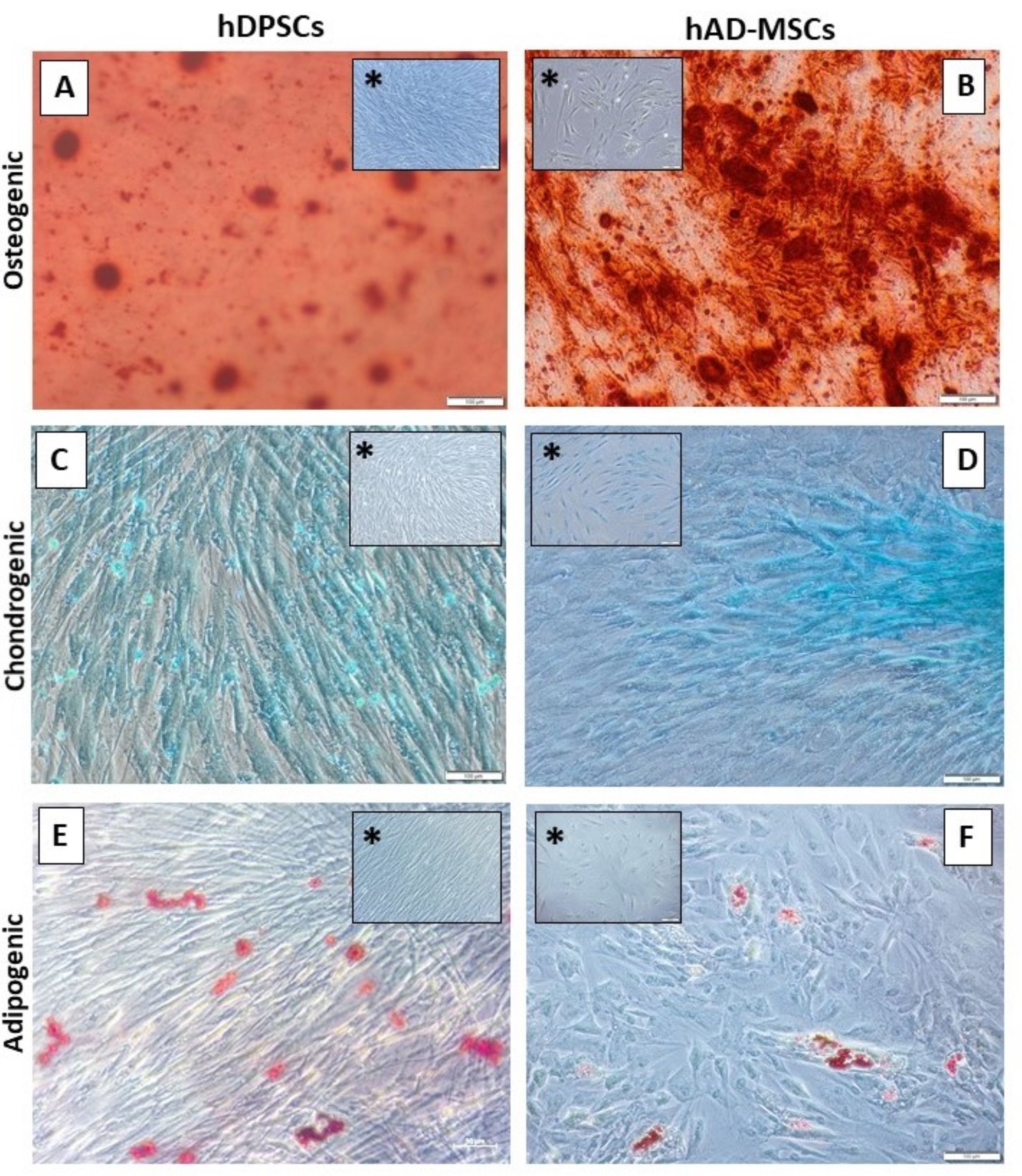

Supplement: Supplementary file 1 [file cells-15-01168-s001.zip › Suppl. 2.jpg]
